# Engineered Toxins “Zymoxins” Are Activated by the HCV NS3 Protease by Removal of an Inhibitory Protein Domain

**DOI:** 10.1371/journal.pone.0015916

**Published:** 2011-01-14

**Authors:** Assaf Shapira, Meital Gal-Tanamy, Limor Nahary, Dana Litvak-Greenfeld, Romy Zemel, Ran Tur-Kaspa, Itai Benhar

**Affiliations:** 1 Department of Molecular Microbiology and Biotechnology, The George S. Wise Faculty of Life Sciences, Tel-Aviv University, Ramat Aviv, Israel; 2 Molecular Hepatology Research Laboratory, Felsenstein Medical Research Center, Sackler School of Medicine, Tel-Aviv University, Petah Tikva, Israel; 3 Department of Medicine D and Liver Institute, Rabin Medical Center, Beilinson Campus, Petah Tikva, Israel; University of Nebraska - Lincoln, United States of America

## Abstract

The synthesis of inactive enzyme precursors, also known as “zymogens,” serves as a mechanism for regulating the execution of selected catalytic activities in a desirable time and/or site. Zymogens are usually activated by proteolytic cleavage. Many viruses encode proteases that execute key proteolytic steps of the viral life cycle. Here, we describe a proof of concept for a therapeutic approach to fighting viral infections through eradication of virally infected cells exclusively, thus limiting virus production and spread. Using the hepatitis C virus (HCV) as a model, we designed two HCV NS3 protease-activated “zymogenized” chimeric toxins (which we denote “zymoxins”). In these recombinant constructs, the bacterial and plant toxins diphtheria toxin A (DTA) and Ricin A chain (RTA), respectively, were fused to rationally designed inhibitor peptides/domains via an HCV NS3 protease-cleavable linker. The above toxins were then fused to the binding and translocation domains of *Pseudomonas* exotoxin A in order to enable translocation into the mammalian cells cytoplasm. We show that these toxins exhibit NS3 cleavage dependent increase in enzymatic activity upon NS3 protease cleavage *in vitro*. Moreover, a higher level of cytotoxicity was observed when zymoxins were applied to NS3 expressing cells or to HCV infected cells, demonstrating a potential therapeutic window. The increase in toxin activity correlated with NS3 protease activity in the treated cells, thus the therapeutic window was larger in cells expressing recombinant NS3 than in HCV infected cells. This suggests that the “zymoxin” approach may be most appropriate for application to life-threatening acute infections where much higher levels of the activating protease would be expected.

## Introduction

The term “zymogen” refers to an inactive enzyme precursor that is converted to its active form following a biochemical modification, usually proteolytic processing. Among the known and important groups of enzymes that are activated in such a way are the cysteine aspartic acid proteases (caspases) which play an essential role at various stages of the apoptotic process [1]; secreted digestive enzymes like pepsin and trypsin [2], [3] and blood coagulating factors [4].

Previously, Alexander Varshavsky suggested the construction of a new kind of toxins referred as “sitoxins”, a concept that artificially combines several functional protein domains to produce a therapeutically effective molecule. More specifically, a sitoxin is comprised of an effector domain, a domain bearing an intracellular signaling moiety; and a polypeptidic sequence located between the effector domain and the domain bearing the intracellular signaling moiety which specifies a cleavage site for a predetermined protease. Following the introduction of the sitoxin into the target cell (that expresses the specific protease), cleavage by the protease separates the effector domain of the sitoxin from the intracellular signaling moiety, resulting either in a longer-lived (and therefore more toxic) effector domain or in an effector domain that moves from a cellular compartment where the domain is nontoxic to a cellular compartment where the domain is able to exert its toxic effect [5]. This strategy was previously applied by Falnes et al., who described the construction of diphtheria toxin based sitoxins that contain a signal for N-end-rule-mediated degradation just upstream of a cleavage site for the protease from HIV type 1. *In-vitro* cleavage by the viral protease substantially increased the ability of the toxins to inhibit cellular protein synthesis; however, the toxins were unable to selectively eradicate HIV-1-infected cells, apparently due to low cytosolic HIV-1 protease activity [6].

*Pseudomonas* exotoxin A (PE) is a three-domain bacterial toxin that kills mammalian cells by gaining entry to the cytosol and inactivating protein synthesis. PE is composed of 3 major domains and 1 minor domain; domain 1a (a.a.1–252) is the cell-binding domain. Domain 2 (a.a.253–364) is the translocation domain that enables PE to reach the cytosol. Domain 3 (a.a.395–613) is the ADP-ribosylating domain that inactivates elongation factor 2 and causes cell death. The pathway of toxin entry includes 1) binding to a surface receptor - PE binds and enters mammalian cells via binding of domain 1 to the alpha 2-macroglobulin receptor/low density lipoprotein receptor-related protein (LRP) which is ubiquitously expressed in most tissues and cell types [7]. 2). Internalization via coated pits to endosomes. 3) Proteolytic cleavage between Arg-279 and Gly-280 within domain 2 and reduction of disulfide bonds [8]. This proteolytic cleavage is mediated by the cellular protease furin, generates the active C-terminal fragment (residues 280–613). 4) Finally, the enzymatically active C-terminal fragment is translocated by retrograde transport through the Golgi apparatus to the endoplasmic reticulum and from there to the cytosol [9], [10], [11]. Once in the cytosol, this fragment inhibits protein synthesis by ADP ribosylating elongation factor 2 [12], [13].

Diphtheria toxin (DT), produced by *Corynebacterium diphtheriae*, kills mammalian cells in a mechanism similar to that of PE, namely, by gaining entry to the cytosol and inactivating protein synthesis by ADP ribosylating elongation factor 2. Diphtheria toxin is composed of three structural and functional domains: the amino terminal catalytic (C) domain (also referred to as “DTA”), the translocation domain (T) and the carboxy-terminal receptor binding (R) domain [14]. DT is cleaved at the surface of sensitive eukaryotic cells by the enzyme furin, and following receptor (the heparin binding epidermal growth factor-like precursor) binding, the di-chain protein that is linked by a single disulfide bond is internalized into clathrin coated pits and reaches the lumen of a developing endosome. Upon acidification, the T domain facilitates the translocation of the catalytic domain directly across the endosomal membrane and into the host cell cytoplasm [15], [16].

*Ricinus communis* ricin toxin is a ribosome-inactivating protein (RIP) which irreversibly damages ribosomes by removal of a single adenine residue (“depurination”) from a GAGA sequence in a universally conserved loop at the top of a stem in 28S rRNA, the so-called “sarcin/ricin loop” (SRL). The toxin consists of two chains linked together by a disulfide bond, one chain of approximately 30 KDa with N-glycosidase enzymatic activity (the A chain), and one of approximately 35 KDa with lectin properties which binds carbohydrate ligands on target cell surface (the B chain) [17]. Cell-bound ricin is taken up by endocytosis. From here, most of the toxin molecules are recycled back to the cell surface or transported to the lysosomes and degraded. Only a small fraction is eventually translocated by retrograde transport to the trans-Golgi network, backward through the Golgi apparatus to the endoplasmic reticulum and from there to the cytosol [18].

HCV is a small, enveloped RNA virus belonging to the *Hepacivirus* genus of the *Flaviviridae* family, which has been recognized as a major cause of chronic liver disease and affects approximately 200 million people worldwide at the present time. Persistent infection is associated with the development of chronic hepatitis, hepatic steatosis, cirrhosis, and hepatocellular carcinoma. A protective vaccine for HCV is not yet available and even the most recent combination of pegylated α-interferon and ribavirin fails to eliminate infection in nearly 50% of those infected [19].

The HCV genome encodes one large open reading frame that is translated as a polyprotein and proteolytically processed to yield the viral structural and nonstructural (NS) proteins. The envelope glycoproteins E1 and E2 as well as the core protein are the structural proteins, which form the viral particle. The non-structural proteins include the p7 ion channel, the NS2-3 protease, the NS3 serine protease/RNA helicase and its co-factor NS4A , the NS4B and NS5A proteins and the NS5B RNA-dependent RNA polymerase (RdRp) [20], [21]. Two virally encoded proteases participate in polyprotein processing, the NS2-3 autoprotease (which cleaves in *cis* at the NS2-3 junction) and the NS3-4A serine protease (also commonly referred to as NS3 protease, which cleaves at four downstream NS protein junctions). NS3 is an extensively studied HCV protein that possesses multiple enzymatic activities that are essential for HCV replication. The N-terminus, in complex with its co-factor NS4A, primarily functions as a serine protease, which cleaves the viral polyprotein precursor downstream to NS3. The remaining 2/3 of the protein has a helicase and NTPase activities, both of which are essential for HCV replication [22].

For a long time, the only approaches to study the full HCV life cycle were experimental infection of chimpanzees, observation of infected patients and comparison with other viruses, members of the *Flaviviridae* family. Recently, different systems enabling the replication and growth of HCV in cell culture (HCVcc) were developed. These advances resulted in the establishment of currently used models to study hepatitis C virus in which recombinant infectious HCV particles (of genotype 2a strain JFH1 and other chimeric viruses generated in the JFH1 background) could be produced in Huh7 hepatoma-derived cell lines that are permissive for HCV replication. HCVcc systems render all steps of the HCV life cycle, including entry and release of viral particles, amenable to systematic analysis. [23], [24], [25], [26], [27], [28], [29], [30], [31].

For the purpose of development of a proof of concept for a potential new anti-viral agents that will specifically eradicate virally infected cells (thus limiting virus production and spread), we designed two chimeric toxins which activation is mediated by HCV-NS3 protease cleavage. These chimeric toxins are based on a fusion between the binding and translocation domains of *Pseudomonas* exotoxin A (serving as a vehicle for translocation into the cytoplasm of target cells) and NS3-activable modified catalytic domains of the bacterial or the plant toxins diphtheria toxin (DTA) or ricin toxin (RTA), respectively. In these constructs, which we denote “zymoxins” (zymogenized toxins), a rationally designed inhibitory peptide/domain is fused to the C terminus of the constitutively active toxins. The inhibitory domain is preceded by the NS3-sensitive NS5A/B junction sequence which cleavage by the viral protease liberates the toxin from the inhibitory moiety. These toxins exhibit considerable level of NS3 protease-dependent activation as was detected by *in-vitro* enzymatic assays, as well as when applied to NS3 expressing model cells (expressing cytosolic or membrane bound NS3). When tested on HCV infected cells, a less dramatic, but still significant enhancement of cytotoxicity was observed, suggesting that eradication of viral protease expressing cells at specific ranges of toxin concentrations can be achieved.

## Results

### Establishment of NS3 protease-expressing model cell system

For the purpose of establishing a model cell line expressing the HCV NS3 protease, we constructed a fusion protein between the previously described NS4A-NS3 (also known as single-chain NS3; scNS3) [32] - a single chain construct where a short synthetic peptide encompassing residues 21–34 of NS4A (of the 1b HCV genotype) was linked to the N terminus of the NS3 protease domain [33], [34], [35], and enhanced green fluorescence protein (EGFP). This format was chosen based on our previous unpublished finding that the expression level of scNS3 in mammalian cells is greatly enhanced upon fusion of efficiently-expressed proteins (such as EGFP) to its N terminus. In addition, another construct was made, comprising a fusion of EGFP and the full length NS3 (protease/RNA helicase) followed by full length NS4A (of the 1a HCV genotype) [36]. As opposed to EGFP-scNS3, which is predicted to be a predominantly cytoplasmic or nucleocytoplasmic protein with relatively low affinity to membranes, the EGFP- full length NS3 (which strongly interacts with the full length NS4A following auto-cleavage) is expected to be associated with intracellular membranes when expressed in mammalian cells, more precisely mimicking the intracellular localization of this complex in HCV infected cells. The reason for that is that the hydrophobic amino terminal domain of the NS4A directs the NS3-NS4A complex to the ER membrane or an ER-like modified compartment [20], [37], [38], [39], [40].

Since signs of cell toxicity were observed following prolonged constitutive expression of EGFP-scNS3 and EGFP-full NS3-4A in HEK293 cells, a TET-ON inducible system for NS3 expression was established. In this system, based on T-REx™ 293 Cell Line (Invitrogen), expression of EGFP-scNS3 or EGFP full NS3-4A (also later referred to as “scNS3” and “full NS3-4A”, respectively) is induced by addition of tetracycline (Tet) to the growth medium.

**Figure 1:**
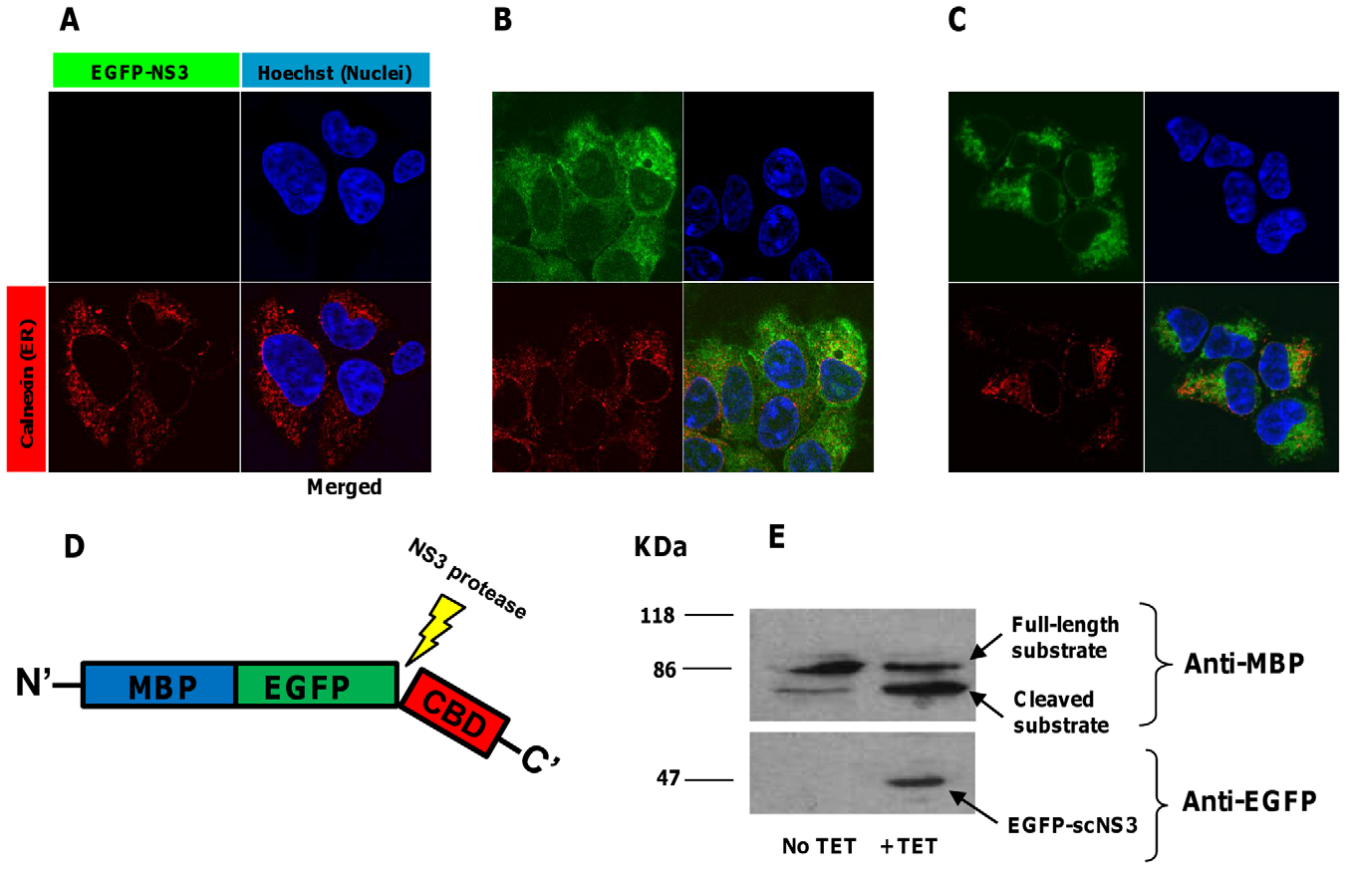
Tetracycline induced expression of active EGFP-scNS3 and EGFP- full NS3-4A in T-Rex 293 cells. Upper panel: immunofluorescence analysis of inducible NS3 expressing cells: 10^5^ cells inducibly expressing EGFP-scNS3 or EGFP-full NS3-4A were seeded on cover-slips in a 24 well-plate. After 12 hours, the cells were treated with 1µg/ml of tetracycline for 24 hours and then were fixed and permeabilized. Following immunostaining with rabbit anti-calnexin and Cy3-conjugated goat anti rabbit for ER visualization (red) and nuclei staining by Hoechst 33258 (Blue), slides were examined by confocal fluorescence microscopy. (**A**). Uninduced cells. (**B**). Induced scNS3 expressing cells. (**C**). Induced full NS3-4A expressing cells. Lower panel: *in-vivo* substrate cleavage by NS3: cells inducibly expressing EGFP-scNS3 were transfected with 2µg of the plasmid pCMV/MBP-EGFP-NS5AB-CBD (“cleavable substrate”- see scheme in **D**). After 24 hours, cells were treated with 1µg/ml of tetracycline or left untreated. 24 hours later, cells were lysed and 20µg protein from total cell extract where analyzed by immunoblotting with rabbit anti-EGFP antibodies (for detection of EGFP-scNS3) and mouse-serum anti-MBP (which is more sensitive than anti-EGFP in detecting the cleavage products of the substrate) followed by HRP-conjugated secondary antibodies and ECL development (**E**). Bands corresponding to the full length substrate, cleaved substrate and EGFP-scNS3 are indicated by arrows. Similar results were obtained also in cells inducibly expressing EGFP-full NS3-A4.

In order to monitor specific NS3 proteolytic activity in Tet induced cells, we constructed a substrate-encoding plasmid that encodes for a modification of our previously described polypeptide which serves as a substrate for proteolysis by the NS3 protease [33]. This plasmid, denoted pCMV/MBP-EGFP-NS5AB-CBD, encodes for a fusion of maltose binding protein (MBP), enhanced green fluorescence protein (EGFP), the 10 amino acid minimal NS3 cleavage sequence (P6-P4′) from HCV NS5A/B site derived from HCV genotype 1b/1a ( for both 1b and 1a genotypes this sequence is identical) [41] and cellulose binding domain (CBD). As shown in Fig. 1, addition of Tet to selected stable clones of the inducible system results in EGFP-scNS3 and EGFP- full NS3-4A expression, as assayed by fluorescence confocal microscopy (Fig. 1B and 1C, respectively) and immunoblotting (Fig. 1E, lower panel). Regarding the intracellular localization of the proteases, EGFP-full NS3-4A was only partially colocalized with calnexin (ER) in these cells, but apparently was absent from the nucleus and has less diffuse distribution pattern in comparison to the nucleocytoplamic EGFP-scNS3, as was expected. A strong colocalization, however, was observed between EGFP-full NS3-4A and a chimeric fluorescent protein which was fused to the C-terminal ER membrane anchor of the tyrosine phosphatase PTP1B [42] (data not shown). Immunoblot analysis also shows efficient cleavage of MBP-EGFP-NS5AB-CBD (referred to as the “cleavable substrate” (Fig. 1D)) in cells transfected with pCMV/MBP-EGFP-NS5AB-CBD and induced for NS3 expression (Fig. 1E). A faint band, corresponding to the cleavage product, was also detected in a sample from uninduced cells. This is probably due to some degree of “leakiness” of the Tet inducible system, allowing a very low basal transcription of the protease in the absence of externally added Tet. In support to this assumption, only a single band corresponding to the full length substrate was detected when “naïve” HEK293 T-Rex cells were transfected with the substrate encoding vector (data not shown).

### The construction of diphtheria toxin based zymoxin

**Figure 2:**
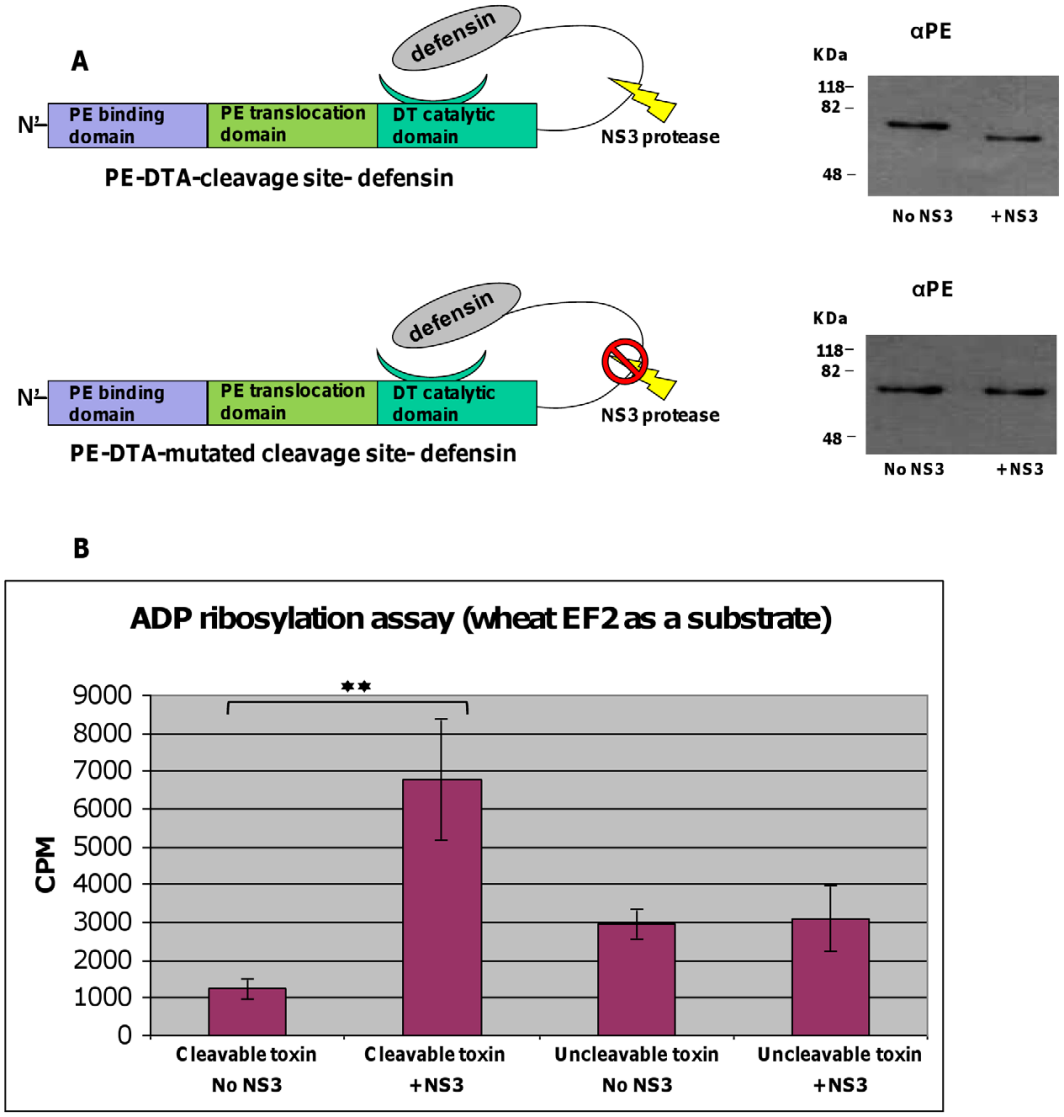
*In-vitro* cleavage by NS3 and ADP-ribosylation assay of DTA-based zymoxins. (**A**). Left panel: a schematic representation of the toxins “PE-DTA-cleavage site-defensin” and “PE-DTA-mutated cleavage site- defensin”. Right panel: *in-vitro* cleavage by NS3: 600ng of each toxin were incubated with or without 500ng of recombinant MBP-scNS3 fusion in a total volume of 60µl for 1 hour at 37°C. Afterwards, 50ng of each toxin were subjected to SDS-PAGE and immunoblotting by anti-PE polyclonal antibodies (αPE) followed by HRP-conjugated goat anti rabbit antibody and ECL development. (**B**). ADP-ribosylation assay for cleaved and uncleaved toxins: 30ng of each toxin (cleaved and uncleaved) were mixed with wheat germ extract and [^14^C]-NAD and incubated for 40 min at RT. Reactions were terminated by addition of TCA to the reaction mixture which resulted in total protein precipitation. Level of ADP-ribosylated EF2 was assessed by measuring the radioactivity of the precipitated protein by a scintillation counter. Results (in CPM) are represented as the mean ±SD of a set of data determined in triplicates (** P-value<0.005).

As can be inferred from toxin-substrate interaction model previously presented by Jorgensen and colleagues, an intimate interaction between DTA and its intracellular target elongation factor 2 is predicted to reside in the C terminal portion of the toxin [43]. Considering that prediction, we assumed that a fusion of a bulky polypeptide moiety to the C terminus of DTA may hinder the toxin-substrate interaction. Furthermore, human alpha-defensin 1 (HNP1) was found to neutralize toxins of the mono-ADP-ribosyltransferase family (to which DTA belongs) [44]. To construct a zymoxin where the catalytic domain of DTA is inhibited by HNP1, and the inhibition is relieved by NS3 cleavage, we chose to fuse HNP1 to the C terminus of DTA. The HNP1 was preceded by the 10 amino acids minimal NS3 cleavage sequence (P6-P4′) from HCV (genotype 1b/1a) NS5A/B site (referred to as “cleavage site”) and a flexible linker of 15 amino acids rich in serine and glycine residues which should allow the HNP1 domain to “fold back” and interact with its interaction site on DTA. This construct was then fused in its N terminus to the binding and translocation domain of *Pseudomonas* exotoxin A (PE) in order to enable internalization and trafficking to the cytosol of mammalian cells (demonstrated previously by Guidi-Rontani [45]), as the natural binding and translocation domains of the diphtheria toxin, which are positioned to the C terminus of the W.T toxin, were substituted by the HNP1 polypeptide. In addition, a 6xHis tag followed by a KDEL ER retrieval signal were placed at the most C terminal portion of the construct (“PE-DTA-cleavage site-defensin”), for facilitating the toxin purification and as the KDEL retrieval system is exploited by PE in order to reach the ER lumen [11], respectively. This 6xHis- KDEL extension was positioned at the C terminus of all following chimeric constructs, which have been expressed in *E. coli* BL21 cells and purified from the periplasmic fraction as described in ‘materials and methods’. As a control, an uncleavable chimeric toxin was constructed, in which the NS3 cleavage site was mutated by substituting P1 cysteine to arginine and P4′ tyrosine to alanine (“PE-DTA-mutated cleavage site- defensin”, see scheme in Fig. 2A). Protein sequences of the zymoxins described above, as well as all the zymoxins used in the study (that will be described later), can be found in supporting Text S1.

### The enzymatic activity of DTA-based zymoxin is elevated following NS3 protease cleavage

In order to evaluate the susceptibility of the above constructs to cleavage by the NS3 protease and to evaluate the influence of such cleavage on their ADP-ribosylation activity, an *in-vitro* cleavage reaction was carried out (by incubating the chimeric toxins with recombinant MBP-scNS3 fusion produced in *E. coli*. [32]), followed by ADP-ribosylation activity assay using wheat germ extract as a source of elongation factor 2 [46], [47]. A schematic representation of the PE-DTA chimeric toxins, the *in-vitro* cleavage products and the ADP-ribosylation assay results are shown in Fig. 2. As shown, incubation of the toxin “PE-DTA-cleavage site-defensin” with the recombinant protease resulted in a complete cleavage of the chimeric toxin that appeared as a lower weight product in immunoblot assay using anti-PE antibodies (Fig. 2A-right and upper panel). Moreover, the cleavage led to a considerable increase in the ADP-ribosylation activity of the toxin (Fig. 2B).

In contrast, the toxin with the mutated cleavage site was resistant to cleavage by NS3, and no significant increase in its ADP-ribosylation activity could be detected after incubation with the protease. Interestingly, the ADP-ribosylation activity of the uncleavable toxin is higher than the basal activity of the uncleaved-cleavable toxin (but lower than the cleaved toxin). However, it should be emphasized at this point that as enzymatic activity and cytotoxicity may be affected by multiple factors such as translocation efficiency, thermodynamic stability, intracellular half-life and interaction with substrate (that might be influenced by even a slight change in the primary sequence of the toxin), basically the most correct control for each cleavable toxin is itself when uncleaved.

### The cytotoxicity of DTA-based zymoxin is elevated in NS3 expressing cells

**Figure 3:**
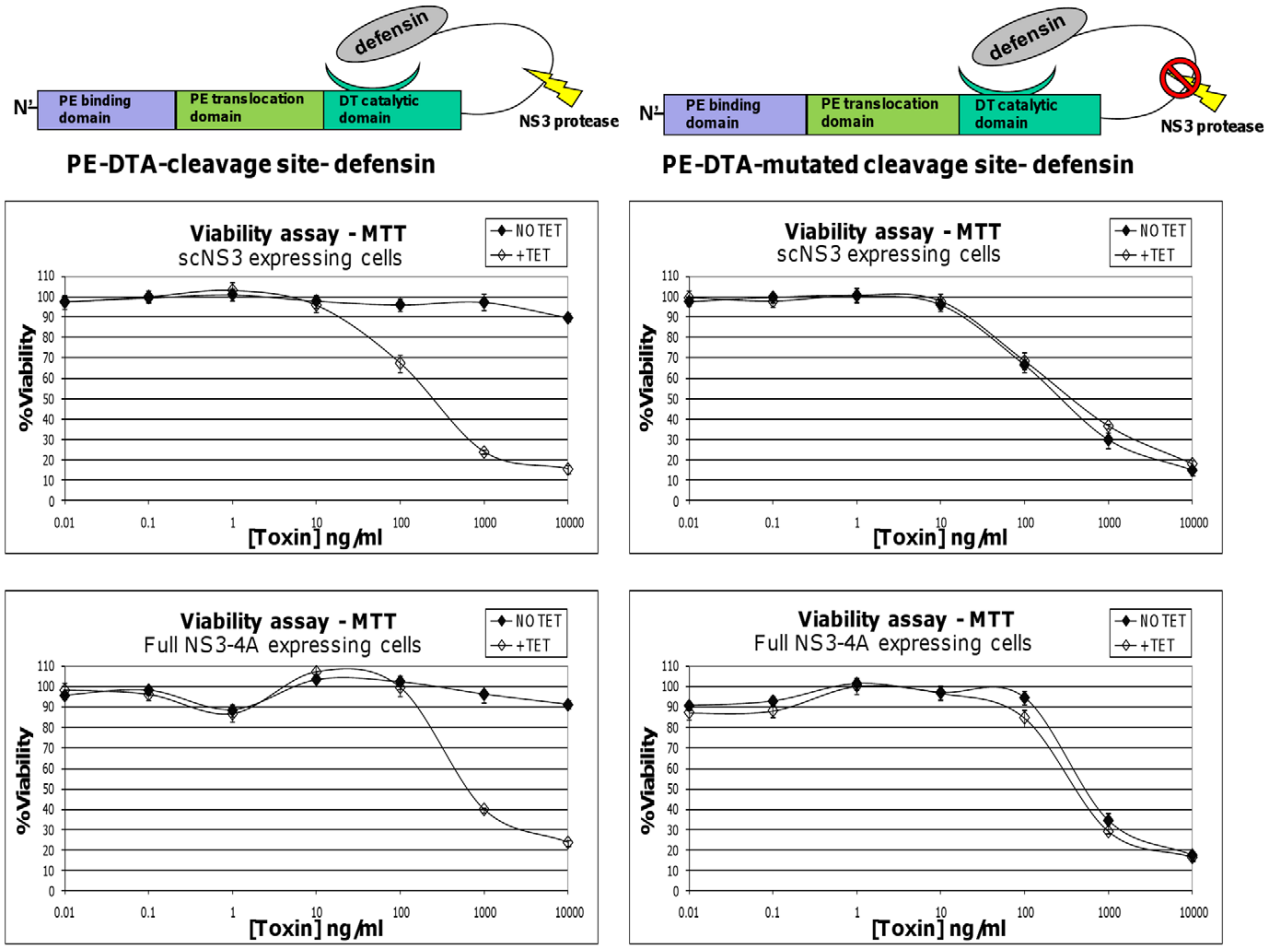
Viability assay of NS3 expressing cells treated with DTA based zymoxins. T-Rex cells inducibly expressing scNS3 or full NS3-4A were seeded in 96-well plates (4×10^4^ cells per well). After 9 hours, cells were treated with 1µg/ml of tetracycline (+TET) or left untreated (NO TET). 2 hours later, cells were incubated for 72 hours with serial dilutions of the toxins “PE-DTA-cleavage site-defensin” or “PE-DTA-mutated cleavage site- defensin” (presence of tetracycline was kept in the growth media of induced cells). The relative fraction of viable cells was determined using an enzymatic MTT assay. A representative graph of three independent experiments is shown. Each point represents the mean ±SD of a set of data determined in triplicates.

In order to evaluate the toxin activation by HCV protease *in-vivo*, our model cell lines, induced or uninduced for NS3 expression, were treated with “PE-DTA-cleavage site-defensin” or “PE-DTA-mutated cleavage site- defensin”. As shown in Fig. 3., tetracycline induction for expression of scNS3 and full NS3-4A has led to considerable activation of the cleavable toxin which presented only a minor cytotoxic effect against uninduced cells. On the contrary, no enhancement in cytotoxic activity of the uncleavable toxin was evident; indicating that induction of NS3 expression does not cause a general sensitization to DTA based toxins in these cells. In agreement with the *in-vitro* ADP-ribosylation assay results, the uncleavable toxin basal activity was more potent than the basal activity of the non-cleaved cleavable toxin (uninduced cells treated with “PE-DTA-cleavage site-defensin”).

In this context, it is worth mentioning that another uncleavable toxin, “PE-DTA-no cleavage site-defensin” in which the whole NS3 cleavage site was deleted, is also indifferent to the presence of the protease and it is as potent as the non-cleaved cleavable toxin (presents only a minor cytotoxic effect against induced or uninduced NS3 expressing cells) (data not shown). These observations further suggest that the most correct evaluation of the therapeutic window a zymoxin may offer is to compare the cleaved to the un-cleaved zymoxin itself.

### The construction of ricin toxin based zymoxin

Since the enzymatic activity of ricin is based on its interaction with ribosomes, one can assume that interruption of this interaction will probably reduce its toxicity. In the recent years, several groups have investigated the nature of the this interaction and it was found that the conserved C terminus of the ribosome stalk proteins P1 and P2 interacts with both ricin and Shiga-like toxin 1 (SLT-1), another type II ribosome-inactivating protein, and this interaction is required for efficient ribosome binding and cytotoxicity. Moreover, a synthetic peptide corresponding to the sequence of the conserved C terminus of P1 and P2 was shown to inhibit the ribosome-inactivating function of SLT-1 [48], [49], [50]. Later on, the crystal structure of a similar peptide in complex with trichosanthin (TCS), a type I ribosome-inactivating protein (RIPs), was presented [51]. Based on these studies, we have cloned the coding sequence of the catalytic A chain of ricin (RTA), originated from *Ricinus communis* genomic DNA preparation, into a bacterial expression plasmid. Next, we have constructed a chimeric toxin in which two repeats of the acidic 16 residue peptide corresponding to the conserved C terminus of the ribosomal stalk proteins (EESEESDDDMGFGLFD) were fused to the C terminus of RTA. Similarly to the concept that was applied to diphtheria toxin chimera described earlier, in the cleavable toxin (“PE-RTA-cleavage site-stalk peptide”) the NS3 protease minimal cleavage sequence (P6-P4′) from genotype 1b/1a NS5A/B junction was inserted between RTA and the ribosome stalk peptide, while in the control uncleavable toxin (“PE-RTA- mutated cleavage site-stalk peptide”) a mutated cleavage site was positioned at this location. This construct was then fused in its N terminus to the binding and translocation domain of PE, as the natural binding and translocation domain of ricin toxin (RTB) undergoes N-glycosylation when produced in *Ricinus communis*, which is necessary for its lectin activity. By fusing RTA to the bacterially derived binding and translocation domains of PE, chimeric PE-RTA toxins that are capable of penetrating into the mammalian cell cytoplasm (demonstrated previously by Pitcher *et al* [52]) may be produced in *E. coli* by standard methods.

### The enzymatic activity of RTA-based zymoxin is elevated following NS3 protease cleavage

**Figure 4:**
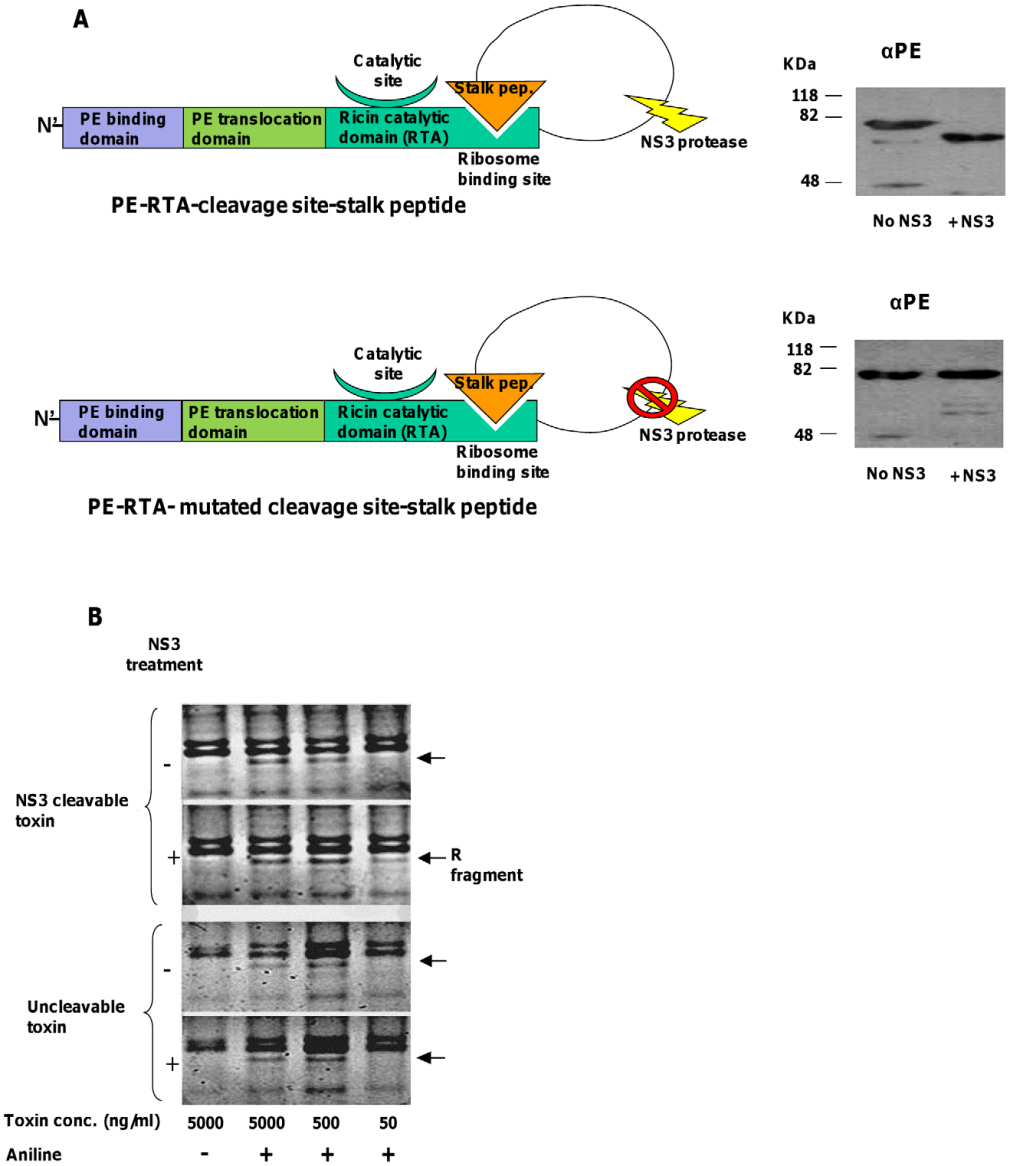
*In-vitro* cleavage by NS3 and ribosome depurination assay of RTA-based zymoxins. (**A**). Left panel: A schematic representation of the toxins “PE-RTA-cleavage site-stalk peptide” and “PE-RTA- mutated cleavage site-stalk peptide”. Right panel: *in-vitro* cleavage by NS3: 3000ng of each toxin were incubated with or without 1000ng of recombinant MBP-scNS3 fusion in a total volume of 60µl for 1 hour at 37°C. Afterwards, 250 ng of each toxin were subjected to SDS-PAGE and immunoblotting by anti-PE (αPE) polyclonal antibodies. (**B**). Ribosome depurination assay: A serial dilution of each toxin (treated or untreated with NS3) was incubated with 10µl of rabbit reticulocyte lysate for 30 minutes at 30°C, after which total RNA from each mixture was extracted. Half of the RNA was treated with acidic aniline (1M aniline in 2.8 M acetic acid) for 10 minutes at 40°C and the other half remained untreated. Next, RNA was extracted and analyzed by 3% TBE agarose gel electrophoresis and staining with ethidium bromide. The position of the “R fragment”, which released from rRNA of ricin-treated ribosomes after treatment with aniline, is marked by an arrow.

In order to assess the susceptibility of the RTA-zbased constructs to cleavage by NS3 and evaluate the influence of such cleavage on their ribosome depurination activity, an *in-vitro* cleavage reaction was carried out (as described for the diphtheria toxin chimera) following by ribosome depurination assay using the “acidic aniline” method on ribosomes from reticulocyte lysate [53], [54]. In this method, the phosphodiester bond at the 3′ site of the depurinated adenine in the ricin treated rRNA is cleaved under treatment with aniline under acidic conditions, and a small fragment of about 460 nucleotides (“R fragment”) is released and can be detected by agarose or acrylamide gel electrophoresis and staining with ethidium bromide. A schematic representation of the PE-RTA chimeric toxins, the *in-vitro* cleavage and the ribosome depurination assay results are represented in Fig. 4.

As shown, incubation of the toxin “PE-RTA-cleavage site-stalk peptide” with recombinant NS3 protease resulted in a complete cleavage of the chimeric toxin that appeared as a lower weight product (Fig. 4A-right and upper panel). In contrast, the uncleavable toxin remained indifferent to the presence of the protease. Moreover, the cleavage has led to an increase in ribosome depurination activity of the toxin, as inferred from the appearance of the “R fragment” when ribosomes were treated with 50 ng/ml of the cleaved toxin. This fragment was undetectable when ribosomes were treated with the same concentration of non-cleaved cleavable toxin (cleavable toxin without incubation with NS3) or with the uncleavable toxin (Fig. 4B). At higher toxin concentrations and in the presence of aniline, the R fragment could be detected after treatment with both the cleaved and the uncleavable toxins. This may result from the fact that the zymoxin is partially and not totally inhibited by the C-terminal extension (as can be also inferred from the cytotoxicity assays shown below).

### The cytotoxicity of RTA-based zymoxin is elevated in NS3 expressing cells

**Figure 5:**
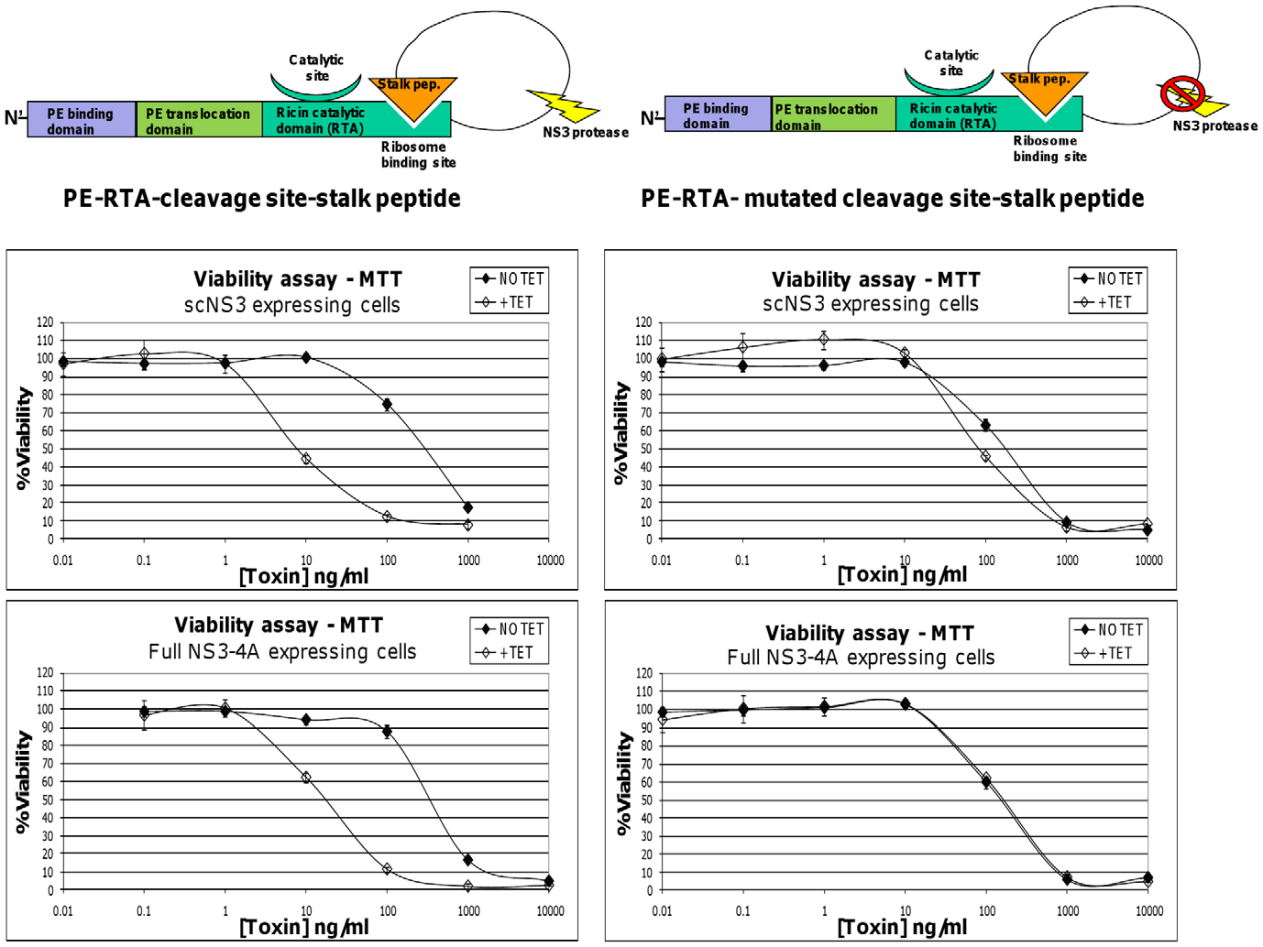
Viability assay of NS3 expressing cells treated with RTA based zymoxins. T-Rex cells inducibly expressing scNS3 or full NS3-4A were seeded (4×10^4^ cells per well) in 96-well plates. After 9 hours, cells were treated with 1µg/ml of tetracycline (+TET) or left untreated (NO TET). Two hours later, cells were incubated for 72 hours with serial dilutions of the toxins “PE-RTA-cleavage site-stalk peptide” or “PE-RTA- mutated cleavage site-stalk peptide” (presence of tetracycline was kept in the growth media of induced cells). The relative fraction of viable cells was determined using an enzymatic MTT assay. A representative graph of three independent experiments is shown. Each point represents the mean ±SD of a set of data determined in triplicates.

In order to verify the activation of the RTA-based toxins by HCV protease *in-vivo*, the model cell lines, induced or uninduced for NS3 expression, were treated with “PE-RTA-cleavage site-stalk peptide” or “PE-RTA- mutated cleavage site-stalk peptide”. As shown in Fig. 5., tetracycline induced expression of scNS3 and full NS3-4A has led to considerable activation of the cleavable toxin, while no profound enhancement in cytotoxicity of the control uncleavable toxin was observed. Another construct, in which only the 6xHis-KDEL extension was fused to the C terminus of RTA, was also relatively inert to scNS3 protease expression exhibiting only minor enhancement in cytotoxicity toward Tet-induced scNS3 expressing cells (IC_50_ values of 30ng/ml vs. 20ng/ml in uninduced and induced cells, respectively) (data not shown).

### The elevated cytotoxicity of DTA and RTA based zymoxins corresponds to the level of intracellular NS3 proteolytic activity

**Figure 6:**
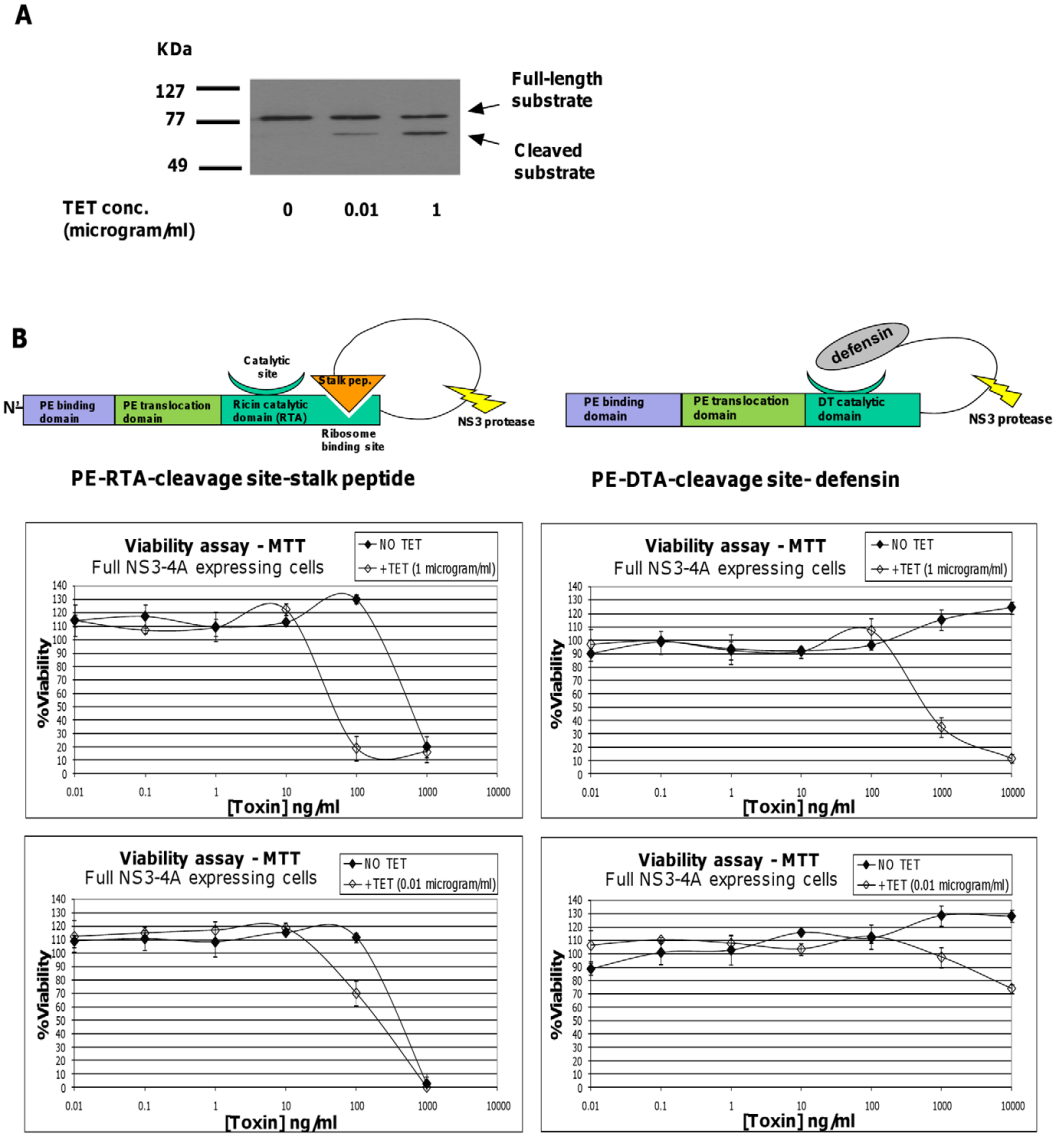
Tetracycline dose-dependence of substrate cleavage efficiency and of zymoxin-treated NS3 expressing cells viability. (**A**). *In vivo* substrate cleavage efficiency analysis: T-Rex cells inducibly expressing full NS3-4A were transfected with 2µg of the vector “pCMV/MBP-EGFP-NS5AB-CBD”. After 24 hours, cells were treated with 1µg/ml of tetracycline, 0.01µg/ml of tetracycline or left untreated. 24 hours later, cells were lysed and samples of 6µg protein from total cell extract where analyzed by immunoblotting with mouse-serum anti-MBP followed by HRP-conjugated secondary antibodies and ECL development. (**B**). Viability assay: T-Rex cells inducibly expressing full NS3-4A were seeded (2×10^4^ cells per well) in 96-well plates. After 24 hours, cells were supplemented with 1µg/ml of tetracycline (+TET (1 microgram/ml)), 0.01µg/ml of tetracycline (+TET (0.01microgram/ml)) or left untreated (NO TET). Eight hours later, cells were incubated for 72 hours with serial dilutions of the toxins “PE-DTA-cleavage site-defensin” or “PE-RTA- cleavage site-stalk peptide” (toxins were diluted in growth media supplemented with the appropriate tetracycline concentration. presence of tetracycline was kept in the growth media of induced cells). The relative fraction of viable cells was determined using an enzymatic MTT assay. Each point represents the mean ±SD of a set of data determined in triplicates.

The elevated cytotoxic effect that was observed in zymoxins treated NS3 expressing cells suggests the *in-vivo* NS3 dependent activation of the zymoxins. Thus, a direct correlation between the level of cellular NS3 proteolytic activity and enhancement in cytotoxicity upon zymoxins treatment is expected. In order to test this assumption, we took advantage of the partially “tunable” nature of the tetracycline inducible NS3 expressing model cell lines system. As shown in Fig. 6A, when T-Rex cells inducibly expressing full NS3-A4 were supplemented with two tetracycline concentrations of 1µg/ml or 0.01µg/ml, a considerably lower *in-vivo* proteolytic activity was observed in the cells supplemented with the lower tetracycline concentration. In agreement, less profound enhancement in cytotoxicity was observed when low tetracycline concentration supplemented cells where treated with both DTA and RTA based zymoxins, (Fig. 6B), demonstrating the expected positive correlation between cellular NS3 proteolytic activity level and enhancement in zymoxins induced cytotoxicity.

### The cytotoxicity of DTA and RTA based zymoxins is elevated in a hepatoma-derived cell line stably expressing NS3 protease or infected with HCV

**Figure 7:**
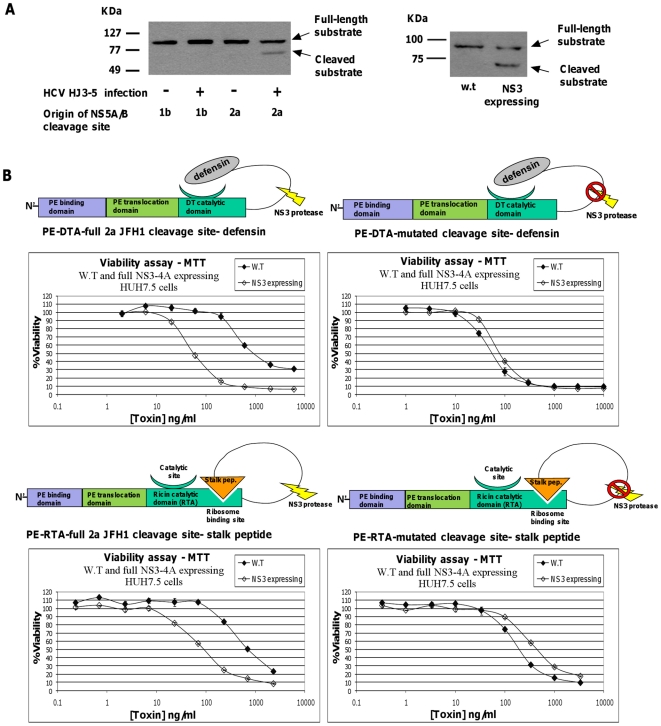
Substrate cleavage efficiency analysis and viability assay of NS3 expressing Huh7.5 cells treated with zymoxins. (**A**). *In vivo* substrate cleavage efficiency analysis. Left panel: Huh7.5 cells uninfected or infected with 1a/2a chimeric virus HJ3-5 were seeded (3×10^5^ cells per well) in 6 well plate. After 24 hours, cells were transfected with 2µg of the vector “pCMV/MBP-EGFP-full 1b NS5AB-CBD” or “pCMV/MBP-EGFP-full 2a JFH1 NS5AB-CBD” incorporating NS3 P10-P8′ and P10-P10′ cleavage sites from 1b genotype or 2a genotype (strain JFH1) NS5A/B junctions, respectively. 48 hours post transfection, cells were lysed and samples of 20–60 µg protein from total cell extracts (normalized to contain similar quantities of total cleavage substrate by evaluation of transfection efficiency using fluorescence microscopy analysis) where analyzed by immunoblotting with mouse-serum anti-MBP. Right panel: w.t Huh7.5 cells or Huh7.5 cells stably and constitutively expressing EGFP-full NS3-4A were seeded (3×10^5^ cells per well) in 6 well plate. After 24 hours, cells were transfected with 2µg of the vector “pCMV/MBP-EGFP-full 2a JFH1 NS5AB-CBD”. 48 hours post transfection, cells were lysed and samples of 15 µg protein from total cell extracts where analyzed by immunoblotting with mouse-serum anti-MBP followed by HRP-conjugated secondary antibodies and ECL development. (**B**). Viability assay: w.t or EGFP-full NS3-4A expressing Huh7.5 cells were seeded (1×10^4^ cells per well) in 96-well plates. After 24 hours, cells were incubated for 96 hours with serial dilutions of the cleavable toxins “PE-DTA-full 2a JFH1 cleavage site-defensin” and “PE-RTA-full 2a JFH1 cleavage site- stalk peptide”, or their uncleavable controls. The relative fraction of viable cells was determined using an enzymatic MTT assay. Each point represents the mean ±SD of a set of data determined in triplicates.

In order to evaluate the potential of the described zymoxins to be cleaved in the cytoplasm of HCV infected cells, Huh7.5 hepatoma cells uninfected or infected with the 1a/2a chimeric virus HJ3-5 (encoding the structural proteins of genotype 1a strain H77S within the background of genotype 2a strain JFH1) [55], [56] were transfected with a new set of cleavage substrates encoding vectors. These vectors, named “pCMV/MBP-EGFP-full 1b NS5AB-CBD” and “pCMV/MBP-EGFP-full 2a JFH1 NS5AB-CBD”, encodes for the previously described NS3 cleavable substrate, with the following modifications: the 10 amino acid minimal NS3 cleavage sequence from genotype 1b/1a NS5A/B junction was replaced by longer cleavage sequences of 18 amino acids (P10-P8′) and 20 amino-acids (P10-P10′) from 1b or 2a (strain JFH1) genotype NS5A/B junction sites, respectively ( longer recognition sequences were demonstrated to be cleaved more efficiently by NS3 [57]). Western blot analysis of lysate samples from uninfected and HJ3-5 infected cells indicates the cleavage of the substrate incorporating the 2a NS5A/B site in infected cells (in which the NS3 protease encoded by the 2a genotype component of the chimera is expressed) (Fig. 7A, left panel). Accordingly, for the following experiments , the 1b/1a genotype derived NS5A/B minimal cleavage site (P6-P4′) was replaced by the longer 2a genotype (strain JFH1) NS5A/B junction sequence (P10-P10′) in both DTA and RTA based cleavable zymoxins (“PE-DTA-full 2a JFH1 cleavage site-defensin” and “PE-RTA-full 2a JFH1 cleavage site- stalk peptide”, respectivelz).

Since HCVcc systems are based on the Huh7.5 hepatoma cell line, we first evaluated whether NS3 expression within Huh7.5 cells would render them sensitive to zymoxin intoxication as was observed for the inducible NS3 expressing HEK293 model cell lines. To this end, Huh7.5 hepatoma-derived cell line stably and constitutively expressing the described EGFP-full NS3-4A (from 1a genotype) was established. These cells were then verified for *in-vivo* NS3 proteolytic activity toward the cleavage substrate encoded by the vector “pCMV/MBP-EGFP-full 2a JFH1 NS5AB-CBD”. As shown in the right panel of Fig. 7A, the substrate was indeed cleaved by the 1a genotype full NS3-A4 protease expressed in these cells. At the Next step, wild-type and EGFP-full NS3-4A expressing Huh7.5 cells were treated with the cleavable zymoxins “PE-DTA-full 2a JFH1 cleavage site-defensin” and “PE-RTA-full 2a JFH1 cleavage site- stalk peptide” , or their uncleavable controls. As shown in Fig.7B, the uncleavable control toxins showed no NS3-dependent enhancement in cytotoxicity. In contrast, toxicity of both cleavable zymoxins was enhanced upon treatment of full NS3-4A expressing Huh7.5 cells, demonstrating that NS3 expression is sufficient for sensitizing hepatoma-derived Huh7.5 cells to these toxins and further emphasizes the potential of these engineered toxins in eradication of NS3 expressing liver cells.

**Figure 8:**
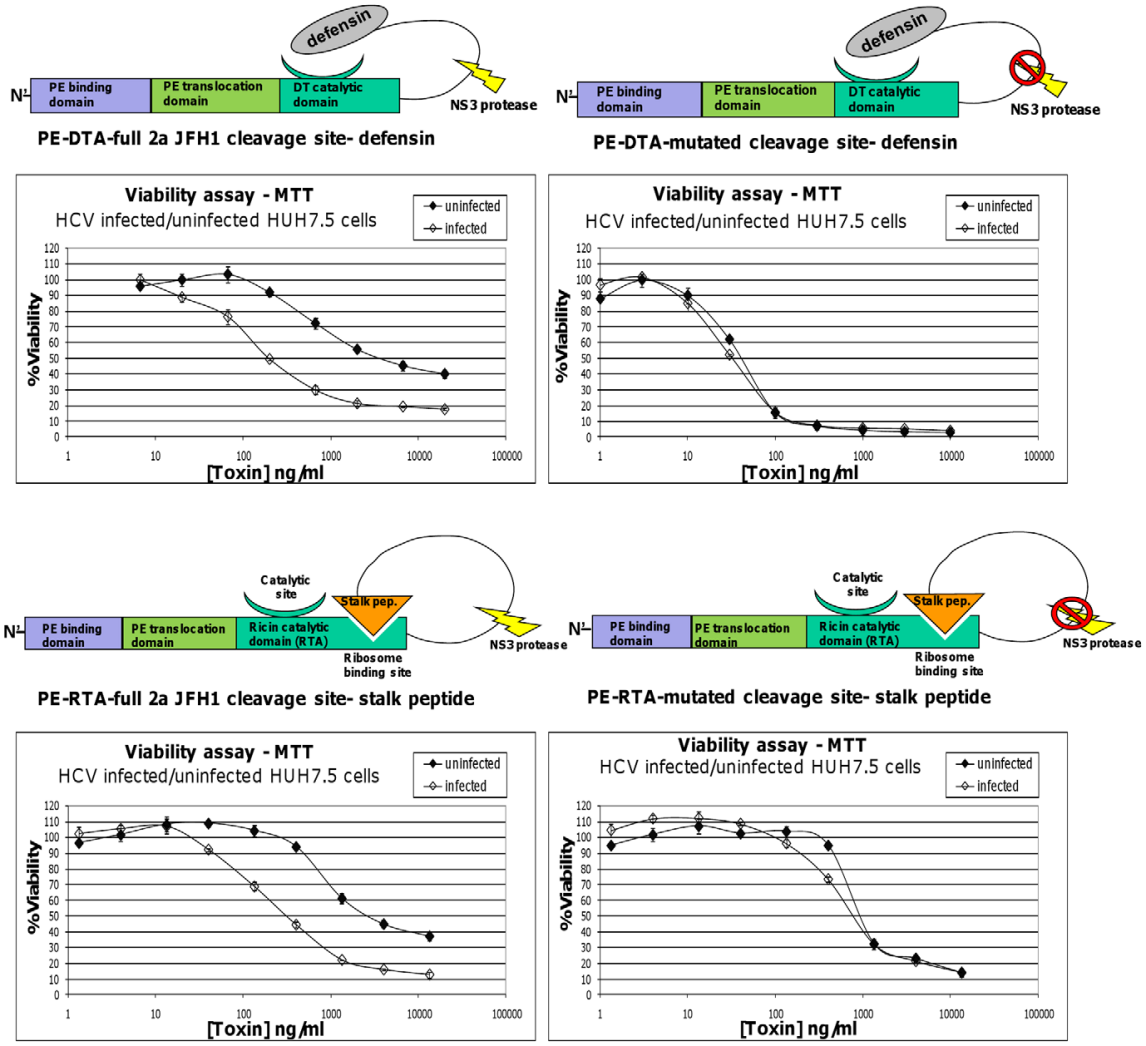
Viability assay of HCV infected/uninfected Huh7.5 cells treated with zymoxins. Huh7.5 cells uninfected or infected with HJ3-5 chimeric virus were seeded (1×10^4^ cells per well) in 96-well plates. After 24 hours, cells were incubated for 96 hours with serial dilutions of the cleavable toxins “PE-DTA-full 2a JFH1 cleavage site-defensin” and “PE-RTA-full 2a JFH1 cleavage site- stalk peptide”, or their uncleavable controls. The relative fraction of viable cells was determined using an enzymatic MTT assay. A representative graph of three independent experiments is shown. Each point represents the mean ±SD of a set of data determined in triplicates.

Next, we turned to evaluate the potential of the described zymoxins in eradicating HCV infected Huh7.5 cells. As can be seen in Fig. 8, treatment of infected/uninfected Huh7.5 cells with both cleavable DTA and RTA based zymoxins resulted in enhanced cytotoxicity upon infected cells (IC_50_ values of ∼3500 and 200 ng/ml for the DTA based toxin toward uninfected and infected cells, respectively; and ∼2500 and 300 ng/ml for the RTA based toxin toward uninfected and infected cells, respectively). Treatment with the uncleavable controls resulted in a much less profound effect (IC_50_ values of ∼40 and 30 ng/ml for the uncleavable DTA based toxin toward uninfected and infected cells, respectively, and ∼900 and 800 ng/ml for the uncleavable RTA based toxin toward uninfected and infected cells, respectively). No enhancement in cytotoxicity upon infected cells was observed following treatment with the DTA-based uncleavable toxin, “PE-DTA-no cleavage site- defensin”, in which the whole NS3 cleavage site was deleted (data not shown). As expected, treatment of infected cells with toxins incorporating NS5A/B cleavage site derived from the 1b genotype (that is inefficiently cleaved by the 2a genotype encoded protease) yielded much reduced enhancement in cytotoxicity, if any (data not shown).

## Discussion

Until these days, vaccination is considered to be the most efficient method for fighting viral infections. However, active vaccination is generally taken as a prevention act and usually when the patient is still uninfected. Moreover, for some viral pathogens which cause world-wide health problem like human immunodeficiency virus (HIV) and HCV, no efficient vaccine had yet been developed.

Intensive efforts over the past decade has been focused on the discovery of anti-viral agents that interfere with specific steps in the viral life cycle which includes viral entry, RNA translation and post-translational processing, reverse transcription, genome integration, viral assembly and release [58], [59], [60]. The processing of a polyprotein precursor by a viral-encoded protease is an essential step in the life cycle of many viruses. A partial list of human diseases associated viruses encoding protease(s) in their genome include flaviviruses such as hepatitis C virus (HCV), West Nile virus (WNV), dengue fever virus (DFV) and yellow fever virus (YFV); retroviruses such as HIV-1, picornaviruses such as coxsackievirus, poliovirus and hepatitis A virus , nidoviruses such as coronaviruses (CoV), including the severe acute respiratory syndrome (SARS) causative SARS-CoV and herpesviruses such as varicella-zoster virus (VZV) and Epstein-Bar virus (EBV) [61], [62]. It is conceivable that many viruses from that list can be targeted by the “zymoxin” approach, depending on the intracellular location and expression/activity levels of their encoded proteases.

Varshavsky suggested an innovative concept for antiviral therapy based on the idea of taking advantage of a specific viral activity instead of inhibiting it. The proposed antiviral agents, which he denoted “sitoxins” (for “signal-regulated cleavage-mediated toxins”), were artificially composed of an intracellular signaling moiety that inactivates a toxin, e.g. a degradation signal, which can be cleaved off by a viral protease, resulting in a selective intoxication of virally infected cells [5]. In later studies, a slightly different strategy for construction of viral-protease activated agents was also applied. This strategy was based on a “zymogenization” of a potentially toxic enzyme, namely, converting a constitutively active enzyme to a protease-activatable form in a way which is not mediated by an intracellular signaling moiety. Such “zymogenized” agents were presented in three elegant studies in which *Plasmodium falciparum*, HIV and HCV protease activated zymogens were designed by circular permutation of bovine RNase A. the enzyme was modified to expose its natively structured active site only upon proteolytic cleavage specifically mediated by the above proteases, which elicited a profound enhancement in *in-vitro* RNase activity [63], [64], [65]. In another study that was carried out by Jucovic and colleagues, a random propeptide library was fused to the C-terminal end of Vip2, an actin modifying vegetative insecticidal protein (Vip) produced by the spore-forming bacterium *Bacillus cereus*. By selecting for malfunctional fusion variants in yeast, they identified a Vip2 proenzyme with significantly reduced enzymatic activity and with the ability to be expressed as a transgene in corn plants without causing developmental pathology. Moreover, the engineered zymogen was still powerful enough to cause the mortality of western corn rootworm larvae, a major pest of corn in the United States, due to proteolytic activation in the rootworm digestive system [66]. Recently, Law *et al* have demonstrated the conversion of a natural zymogen, the maize ribosome inactivating protein (maize RIP), into an HIV protease-activated toxin [67]. The maize RIP is a type III ribosome-inactivating protein which in its native form is synthesized as inactive precursor (pro-RIP) and is activated by proteolytic removal of a 25 amino-acid inhibitory internal peptide *in-planta* [68], [69]. By replacing the first and the last 10 residues of the internal inactivation region with two HIV-protease recognition sequences, a modified zymogen toxin was prepared which showed enhanced enzymatic and anti-viral activity (it was demonstrated that several RIPs possess a potent antiviral activity [70]) in HIV infected cells [67]. However, no data relating to differences between the cytotoxicity against uninfected vs. infected cells was provided; thus the potency of this re-engineered toxin in actual eradication of HIV infected cells cannot be determined. When treating retroviral infection, eradication of infected cells has an important “added value” over inhibition of viral replication and particle production in living cells. The reason for this is that the infected cells may contain an integrated proviral DNA in their genome, which can only be eliminated by destroying the cell itself. During our zymoxins study described herein, we have tried a similar approach in order to convert the maize RIP into an HCV protease-activated toxin. However, in our hands, insertion or replacement of sequences surrounding the internal inhibitory peptide with NS3 cleavable sequences did not generated a toxin that exhibits significant enhancement in cytotoxicity against NS3 expressing cells (unpublished data).

Integrating the concept of “sitoxins” with the strategy of enzyme “zymogenization”, we have developed two rationally designed viral-protease activated chimeric toxins, which we denoted “zymoxins” for “zymogenized toxins”. The HCV NS3 protease has been chosen to serve as a model for a virally encoded protease which expression and activity in infected cells is essential for viral replication/maturation [22]. In both zymoxins, the binding and translocation domain of *Pseudomonas* exotoxin A (PE) were used as a vehicle for the delivery of NS3 cleavable “zymogenized” catalytic subunits of the diphtheria toxin A (from *Corynebacterium diphtheria*) or ricin A (from *Ricinus communis*) into the cytosol of mammalian cells [45], [52].

In contrast to sitoxins, in which the inhibitory activity is mediated by intracellular components which recognize a cleavable signaling polypeptide fused to a toxin; the inhibition of the toxic activity of the zymoxins is mediated solely by the modification applied to the original toxin. Therefore, we first confirmed NS3 proteolytic activation of the two zymoxins by *in-vitro* activity assays. In order to assay translocation capability and NS3 proteolytic activation *in-vivo*, a model HEK293 cell line inducibly expressing the HCV NS3-protease was established. It was previously reported that sitoxins that are pre-activated *in-vitro* by the HIV protease have increased ability to inhibit cellular protein synthesis. However, these sitoxins subsequently failed to inhibit the growth of HIV infected cells, and it was suggested by the authors that this failure was due to the fact that the HIV virus-encoded protease is located at the plasma membrane and not available to activate the sitoxins [6]. Hence, zymoxins activation was assayed using both cytosolic and membrane-associated NS3 expressing cells as cellular localization of the protease may influence the probability of interaction with translocated toxins. The latter model of membrane associated NS3 expression system was particularly important since it should more precisely mimic the intracellular localization of the genuine viral-encoded NS3 protease domain.

Our results show a considerable NS3-dependent enhancement in cytotoxicity for the two zymoxins in both model cell lines, leading to specific eradication of protease expressing cells at specific ranges of zymoxin concentrations. This demonstrates the competence of the binding and translocation domains of PE to mediate delivery of the DTA and RTA catalytic domains into cells cytoplasm and, importantly, that NS3 cleavage mediated enhancement in the enzymatic activity of the two zymoxins (as was detected by *in-vitro* assays) is practically translated to enhancement in cytotoxic activity.

In order to test the competence of the zymoxins in eradicating HCV infected liver cells, we utilized one of the currently used models to study hepatitis C virus in which recombinant infectious HCV particles are produced in cell culture (HCVcc) based on Huh7.5 hepatoma cell line [23], [24], [25], [26], [27], [28], [29], [30], [31], [56]. First, we have verified that NS3 expression sensitizes Huh7.5 liver cells to zymoxin intoxication as was observed following inducible NS3 expression in HEK293 cell lines. Finally, we show that treatment with both cleavable DTA and RTA based zymoxins resulted in enhanced cytotoxicity toward HCV infected Huh7.5 cells. In contrast, when the cells were treated with uncleavable control toxins, no therapeutic window was observed. Further support to the authenticity of these results was provided by our unpublished observations that toxins incorporating 1b genotype derived HCV NS5A/B site (that is inefficiently cleaved by the 2a genotype encoded protease in HCV infected cells) resulted in a minor or undetectable enhancement in cytotoxicity upon treatment of infected cells.

Of note in the experiments with HCV infected Huh7.5 cells the “therapeutic windows” were up to ∼15 fold, which were narrower than these observed in the case of the NS3 inducibly expressing HEK293 T-Rex cell line under conditions of full induction. This value is also smaller than these observed in similar experiments on NS3 expressing Huh7.5 cells, were therapeutic windows of up to 30 fold were recorded. Apparently, the level of NS3 protease activity in HCV infected cells is lower than the level in the model cell lines, which is also evident when comparing the left and right panels of Fig. 7A.

In conclusion, the design of the two anti-viral agents presented in this work was inspired by the “sitoxins” concept presented by Varshavsky and recent work in the field of enzyme engineering. The ability of these agents to be activated by a viral protease provides a proof of concept for the feasibility of “zymogenization” of natural toxins by rational design relaying on previous knowledge on the toxin's structure, interaction with substrate and existence of peptidic inhibitors. Although practical use may require further research and development in order to broaden the “therapeutic window” and reduce potential immunogenicity of these drugs; eradication of virally infected cells by using zymogenized toxins may represent another strategy in fighting viral diseases. Still, the “zymoxin” approach may be most appropriate for application to life-threatening acute infections where much higher levels of the activating protease than the ones observed in HCV infected cells would be expected.

## Materials and Methods

### Bacterial strains

The following *Escherichia coli* strains were used: XL-1 Blue (Stratagene, USA) for plasmid propagation and Rosetta (DE-3) (Novagen, USA) for expression of the T7 promoter-driven recombinant toxins.

### Recombinant DNA techniques and vectors

Recombinant DNA techniques were carried out according to standard protocols or as recommended by suppliers. Nucleotide sequences were determined using the PRISM 3100 Genetic Analyzer (Applied Biosystems, USA) according to the supplier's recommendations. The bacterial T7 promoter-based expression vector pET28a, used for expression of recombinant toxins in *Escherichia coli*, was from Novagen (USA). The eukaryotic tetracycline-inducible CMV promoter-based expression vector pcDNA 4/TO, used for expression of EGFP-scNS3 and EGFP- full NS3-4A in T-REx 293 Cell Line was from Invitrogen (USA). All plasmid and DNA fragment purifications were carried out with HiYield Plasmid Mini Kit and HiYield Gel/PCR DNA Extraction Kit (RBC bioscience, Taiwan). T4 DNA ligase and restriction enzymes were purchased from New England Biolabs (USA). DNA ligations were carried out at 16°C overnight.

### Plant genomic DNA extraction

Genomic DNA extraction from *Ricinus communis* was performed as described in [71].

### Construction of NS3 protease expression vectors

#### Construction of the tetracycline-inducible vector encoding EGFP-scNS3

the previously described NS4A-NS3 (single-chain NS3; scNS3) [32], [33], [34], [35] coding sequence was amplified by PCR using the plasmid “pMGT14” [32] DNA as template, and the primers 1-cytons3 and 2-cytons3 (primer sequences are given in supporting Table S1).

The PCR product was digested with *Hind*III and *Apa*I (restriction sites are underlined in the primer sequences) and was cloned between the corresponding sites in pEGFP-C2 (Clontech, USA), generating plasmid “pEGFP C2- scNS3”. Next, a fragment containing the coding sequence of the EGFP-scNS3 fusion was excised from the above plasmid by digestion with *Eco47* III and *Apa*I and was cloned between the corresponding sites in the Tetracycline inducible vector pcDNA 4/TO , generating plasmid “pcDNA 4/TO EGFP-scNS3”.

#### Construction of the tetracycline-inducible vector encoding EGFP- full NS3-4A

The coding sequence of the full length NS3 (including the helicase domain) followed by NS4A from 1a HCV genotype was excised from the plasmid “NS3/4A-pVAX1” [36], kindly provided by Prof. Matti Sällberg, Karolinska University Hospital, Sweden, by digestion with *Nar*I and *Apa*I. The DNA fragment was then cloned between the corresponding sites in “pcDNA 4/TO EGFP-scNS3” (replacing the scNS3 sequence), generating plasmid “pcDNA 4/TO EGFP-Full NS3-4A”.

### Construction of vectors for expression of zymoxins based on DT

#### Construction of the vector encoding the “PE-DTA-cleavage site-defensin” zymoxin (shown schematically in Fig. 2A)

The coding sequence of amino acids 1–605 of *Pseudomonas* exotoxin A (PE), including a signal peptide, was amplified by PCR from a plasmid encoding a PE derivative (PE-QQ delta) kindly provided by Dr. Ira Pastan, NCI, NIH, Bethesda, MD, USA, in which lysines 590 and 606 where substituted with glutamine residues and lysine 613 was deleted [72].

In the same amplification process, a DNA sequence encoding for the 10 amino acid minimal NS3 cleavage sequence (P6-P4′) from HCV NS5A/B site derived from HCV genotype 1b/1a [41] followed by *Mfe*I site, *Pst*I site, 6 histidine residues (6xHIS) and the KDEL ER retrieval signal was introduced to the 3′ end of the toxin coding sequence. The primers that have been used are the forward primer 3-DTAclv; And the reverse primers: (in this order, when the PCR product of the forward and the first reverse primer used as template for the reaction with the forward and the second reverse primer, and so on): 4-DTAclv , 5-DTAclv and 6-DTAclv. The final PCR product was digested with *Xba*I and *Eco*RI and was cloned between the corresponding sites in the bacterial expression plasmid pET28a generating the plasmid “pET28a PE QQ delta NS5AB-HIS-KDEL”. Next, the human alpha-defensin 1 (HNP1) coding sequence preceded by a flexible linker of 15 amino acids and followed by a linker of 4 amino acids, both are rich in glycine and serine, was inserted between the NS5AB and the 6xHIS of the above plasmid. This insert was created by PCR using the plasmid “pET28a PE QQ delta NS5AB-HIS-KDEL” DNA as template, the reverse primer 7-DTAclv; And the forward primers: (in this order, when the PCR product of the reverse and the first forward primer used as template for the reaction with the reverse and the second forward primer, and so on): 8-DTAclv, 9- DTAclv, 10- DTAclv and 11- DTAclv.

The PCR product was digested with *Pst*I and *Eco*RI and was cloned between the corresponding sites in the same plasmid that was used as template, generating the plasmid “pET28a PE QQ delta NS5AB-15aa linker-HNP-HIS-KDEL”. In the next step, a DNA fragment encoding diphtheria toxin A (kindly provided by Prof. Nadir Arber, Integrated Cancer Prevention Center, Tel Aviv Sourasky Medical Center, Israel) was used as template for PCR amplification of the region encoding amino acids 1–187 of the mature toxic A domain (without the signal peptide). In the same amplification process, a DNA sequence encoding the 1b derived P6-P4′ NS5A/B junction was introduced to the 3′ end of the toxin coding sequence. The primers that have been used are 12- DTAclv and 13- DTAclv.

The PCR product was digested with restriction enzymes *Aat*II and *Mfe*I and was cloned between the corresponding sites in the plasmid “pET28a PE QQ delta NS5AB-15aa linker-HNP-HIS-KDEL” generating the plasmid “pET28a PE QQ delta -DTA(1–187)(instead domain III) -NS5AB-15aa linker- HNP-HIS-KDEL” in which the catalytic domain of PE (domain III) was replaced by amino acids 1–187 of DTA, followed by the NS3 cleavable NS5A/B junction sequence, a flexible linker of 15 amino acids rich in glycine and serine, the human alpha-defensin 1 (HNP1) coding sequence, a short 4 amino acid linker, 6xHIS and the KDEL retrieval signal. Protein sequences of the zymoxin described above, as well as all the zymoxins used in the study can be found in supporting Text S1.

#### Construction of the vector encoding the control uncleavable zymoxin “PE-DTA-mutated cleavage site- defensin” (shown schematically in Fig. 2A)

The minimal NS3 cleavage sequence (P6-P4′) derived from NS5A/B junction of HCV 1b/1a genotype in the plasmid “pET28a PE QQ delta -DTA(1–187)(instead domain III) -NS5AB-15aa linker- HNP-HIS-KDEL” was mutated by substituting P1 cysteine to arginine and P4′ tyrosine to alanine using the DNA of plasmid “pET28a PE QQ delta -DTA(1–187)(instead domain III) -NS5AB-15aa linker- HNP-HIS-KDEL” as template with the following primers: Forward 14-DTAunc ; and reverse: 15-DTAunc and 16-DTAunc. The PCR product was digested with *Aat*II and *Pst*I and was cloned between the corresponding sites in the same plasmid that was used as template, generating the plasmid “pET28a PE QQ delta -DTA(1–187)(instead domain III)-mutated NS5AB- 15aa linker-HNP-HIS-KDEL”.

#### Construction of the vector encoding the control DTA based uncleavable toxin, in which the whole NS3 cleavage site was deleted (“PE-DTA-no cleavage site- defensin”)

A PCR was carried out using DNA of plasmid “pET28a PE QQ delta -DTA(1–187)(instead domain III) -NS5AB-15aa linker- HNP-HIS-KDEL” as template and the primers 17-DTAnoclv and 18-DTAnoclv. The PCR product was digested with *Pst*I and *Aat*II and was cloned between the corresponding sites in the same plasmid that was used as template, generating the plasmid “pET28a PE QQ delta -DTA(1–187)(instead domain III)- no cleavage site- 15aa linker-HNP-HIS-KDEL ”.

#### Construction of the vector encoding “PE-DTA-cleavage site-defensin” zymoxin in which the P6-P4′ NS3 cleavage sequence derived from 1b genotype NS5A/B junction was replaced by the P10-P10′ cleavage sequence derived from 2a genotype (strain JFH1) NS5A/B junction (“PE-DTA-full 2a JFH1 cleavage site-defensin”)

A PCR was carried out using DNA of plasmid “pET28a PE QQ delta -DTA(1–187)(instead domain III) -NS5AB-15aa linker- HNP-HIS-KDEL” as template, the reverse primer : 19-DTA2aclv; and the forward primers: 20- DTA2aclv and 21- DTA2aclv . The PCR product was digested with *Stu*I and *Xho*I and was cloned between the corresponding sites in a plasmid similar to the one used as template, in which the *Stu*I site was introduced silently (without changing the protein sequence) upstream to the 1b derived NS5A/B sequence, generating plasmid “pET28a PE QQ delta -DTA (1–187) (instead domain III)-full 2a JFH1 NS5AB-15aa linker–HNP-HIS-KDEL”.

### Construction of vectors for expression of zymoxins based on RTA

#### Construction of the vector encoding the RTA based cleavable zymoxin “PE-RTA-cleavage site-stalk peptide” (shown schematically in Fig. 4A)

The coding sequence of the catalytic domain, ricin toxin A chain (amino acids 1–267) was amplified from *Ricinus communis* genomic DNA preparation by PCR using the primers: 22-RTAclv and 23-RTAclv. The PCR product was digested with *Sac*II and *Mfe*I and was cloned between the corresponding sites of plasmid “pET28a PE QQ delta NS5AB-HIS-KDEL”, generating the plasmid “pET28a PE QQ delta (I-II-Ib-RTA)-HIS-KDEL”, which served as template for another PCR using the forward primer: 24-RTAclv; and the reverse primers: 25- RTAclv, 26- RTAclv, 27- RTAclv and 28- RTAclv. The PCR product was digested with *Sac*II and *Pst*I, and was cloned between the corresponding sites in the same plasmid that has been used as template, generating plasmid “pET28a PE QQ delta RTA-NS5AB-short linker-stalk peptide-HIS-KDEL” in which two repeats of the acidic 16 residue peptide corresponding to the conserved C terminus of the ribosomal stalk proteins (EESEESDDDMGFGLFD) have been fused to the C terminus of RTA, preceded by the P6-P4′ NS3 cleavable NS5A/B junction sequence derived from 1b/1a genotype, a short linker of Gly-Gly-Gly-Gly-Ser and followed by 6xHIS and the KDEL ER retrieval signal.

#### Construction of the vector encoding the uncleavable control zymoxin “PE-RTA- mutated cleavage site-stalk peptide” (shown schematically in Fig. 4A)

The minimal NS3 cleavage sequence (P6-P4′) derived from NS5A/B junction of HCV 1b/1a genotype in the plasmid “pET28a PE QQ delta RTA-NS5AB-short linker-stalk peptide-HIS-KDEL” was mutated by substituting P1 cysteine to arginine and P4′ tyrosine to alanine using the DNA of plasmid “ pET28a PE QQ delta RTA-NS5AB-short linker-stalk peptide-HIS-KDEL” as template and the primers : 29-RTAunc and 30-RTAunc. The PCR product was digested with *BamH*I and *EcoR*I, and was cloned between the corresponding sites in the same plasmid that has been used as template, generating the plasmid “pET28a PE QQ delta RTA-mutated NS5AB-short linker-stalk peptide-HIS-KDEL”.

#### Construction of the vector encoding “PE-RTA-cleavage site-stalk peptide” zymoxin in which the P6-P4′ NS3 cleavage sequence derived from 1b genotype NS5A/B junction was replaced by the P10-P10′ cleavage sequence derived from 2a genotype (strain JFH1) NS5A/B junction (“PE-RTA-full 2a JFH1 cleavage site- stalk peptide”)

A PCR was carried out using DNA of plasmid “pET28a PE QQ delta RTA-NS5AB-short linker-stalk peptide-HIS-KDEL” as template, the reverse primer: 31-RTA2aclv; and the forward primers: 32- RTA2aclv and 33- RTA2aclv. The PCR product was digested with *Bam*HI and *Xho*I and was cloned between the corresponding sites in the same plasmid that has was as template, generating the plasmid “pET28a PE QQ delta RTA-full 2a JFH1 NS5AB-short linker-stalk peptide-HIS-KDEL”.

### Construction of the vectors encoding NS3 cleavable substrates

#### Construction of the vector encoding the NS3 cleavable substrate “MBP-EGFP-NS5AB-CBD” bearing the P6-P4′ NS3 cleavage sequence derived from 1b/1a genotype NS5A/B junction (shown schematically in Fig. 1D)

The EGFP-NS5A/B-CBD coding sequence was excised from the plasmid “pYB-44” [33] by digestion with *Nco*I and *Bam*HI, and ligated into the corresponding sites in the plasmid “pCMV/*H6myc*/Cyto-MBP” [73], generating plasmid “pCMV/MBP-EGFP-NS5AB-CBD”.

#### Construction of the vector encoding the NS3 cleavable substrate “MBP-EGFP-full 1b NS5AB-CBD” bearing the P10-P8′ NS3 cleavage sequence derived from 1b genotype NS5A/B junction

A PCR was carried out using DNA of plasmid “pCMV/MBP-EGFP-NS5AB-CBD” as template, the reverse primer: 34-subful1b; and the forward primers: 35- subful1b and 36- subful1b. The PCR product was digested with *Nhe*I and *Hin*dIII and was cloned between the corresponding sites in the same plasmid that was used as template, generating plasmid “pCMV/MBP-EGFP-full 1b NS5AB-CBD”.

#### Construction of the vector encoding the NS3 cleavable substrate “MBP-EGFP- full 2a JFH1 NS5AB-CBD” bearing the P10-P10′ NS3 cleavage sequence derived from 2a genotype (strain JFH1) NS5A/B junction

A PCR was carried out using DNA of plasmid “pCMV/MBP-EGFP-NS5AB-CBD” as template, the reverse primer: 37-subful2a; and the forward primers: 38- subful2a and 39-subful2a. The PCR product was digested with *Nhe*I and *Hin*dIII and cloned between the corresponding sites in the same plasmid that was used as template, generating plasmid “pCMV/MBP-EGFP-full 2a JFH1 NS5AB-CBD”.

### Protein expression and purification

*E. coli* BL21 Rosetta (DE3) cells were transformed with pET28a based expression plasmids and grown in 1 liter of LB medium supplemented with 50 µg/ml Kanamycin, at 37°C 250 rpm shaking to O.D.600nm of 0.8. The cells were chilled down to 30°C and induced with 1mM IPTG for 3–4 hours at 30°C, 250 rpm. The cells were collected by centrifugation at 5000g, at 4°C for 10 minutes. For preparation of periplasmic fractions, the cell pellet was gently re-suspended in 200 ml of ice-cold 20% sucrose, 30mM Tris-HCl (pH 7.4), 1mM EDTA, using glass beads. The cell suspension was incubated on ice for 15 minutes and centrifuged at 6000g, at 4°C for 15 minutes. The cell pellet was gently re-suspended in 200 ml of ice cold sterile double distilled water (DDW), incubated on ice for 15 minutes and centrifuged at 8000g, 4°C 20 minutes. The supernatant periplasmic fraction was adjusted to 20mM Tris-HCl (pH 8.0), 300mM NaCl and 5mM Imidazole. The periplasmic fraction was incubated over-night, in continues rotation with 350µl of Ni-NTA resin (Favorgen, Taiwan) that was previously equilibrated with binding buffer (20mM Tris-HCl pH 8.0, 300mM NaCl). Ni-NTA resin was then separated from the periplasmic supernatant by 5 minutes centrifugation at 70g, 4°C, loaded on Poly Prep column (Bio-Rad, USA) and washed with 20ml of binding buffer +5mM imidazole. Bound His-tagged protein was subsequently eluted with 700µl PBS containing 500mM imidazole, and dialyzed twice against 1 liter of PBS.

### Cell culture, transfection, protein extraction and immunoblotting

Human embryonic kidney cells HEK293, stably expressing the tetracycline repressor protein (T-REx 293 Cell Line, Invitrogen, USA), and human hepatoma cells Huh7.5 [31] were used throughout this study. Cell lines were maintained in DMEM supplemented with 10% fetal calf serum (FCS), 2 mM L-glutamine, 100 U/ml penicillin, 100 µg/ml streptomycin and 12.5 U/ml nystatin (Biological Industries, Israel) in a humidified 5% CO_2_ incubator at 37°C.

The calcium-phosphate transfection method was applied for introducing 2µg of the plasmids “pcDNA 4/TO EGFP-scNS3”, “pcDNA 4/TO EGFP-Full NS3-4A” or “pCMV/MBP-EGFP-NS5AB-CBD” into T-Rex 293 cells, seeded 1.5×10^6^ cells per 6cm plate 24 hours before transfection. Stable transfectants, inducibly expressing EGFP-scNS3 or EGFP-Full NS3-4A were selected in a medium containing zeocin (100µg/ml and 50µg/ml for selection and maintenance, respectively) (CAYLA, France).

2µg of the plasmids “pCMV MBP-EGFP-full 1b NS5AB-CBD”, “pCMV MBP-EGFP-full 2a JFH1 NS5AB-CBD” or “pcDNA 4/TO EGFP-Full NS3-4A” were introduced into uninfected or HCV infected Huh7.5 Cells (seeded 3×10^5^ cells per well in 6-well plate 24 hours before transfection) using FuGENE 6 reagent (Roche, Germany), according to the manufacturer instructions. Stable transfectants, constitutively expressing EGFP-Full NS3-4A were selected and maintained in a medium containing zeocin (100µg/ml and 5µg/ml for selection and maintenance, respectively).

For protein extraction, 48 hours post-transfection the cells were washed with PBS, scraped and lysed in a buffer containing 150 mM NaCl, 5 mM EDTA, 0.5% NP-40, 10 mM Tris(HCl) pH 7.5, and protease inhibitors cocktail (Sigma, Israel). Following 30 minutes of incubation on ice, lysates were cleared by centrifugation at 20,000 *g* for 10 minutes, at 4°C. For immunoblotting, protein samples were electrophoresed on 12% SDS/polyacrylamide gel, transferred to nitrocellulose and detected using rabbit polyclonal anti-GFP antibody (Santa-Cruz, USA) or polyclonal mouse serum anti-MBP followed by HRP-conjugated goat anti-rabbit or anti-mouse mouse antibodies (Jackson ImmunoResearch Laboratories, USA) and ECL detection. Immunoblotting of purified recombinant toxins was similarly performed, using rabbit polyclonal anti-PE antibody, kindly provided by Dr. Ira Pastan, NCI, NIH, Bethesda, MD, USA.

### Viral infection

Virus assays were carried out with an inter-genotypic chimeric virus produced by replacing the core-NS2 segment of the JFH-1 virus genome with the comparable segment of the genotype 1a H77 virus. This chimeric virus, HJ3-5 (Kindly provided by Prof. Stanley Lemon, University of Texas at Galveston), contains two compensatory mutations that promote its growth in cell culture as described previously [55], [56]. HCV RNAs were transcribed *in vitro* and electroporated into cells essentially as described previously [74], [75]. In brief, 10 µg of *in vitro*-synthesized HCV RNA was mixed with 5×10^6^ Huh7.5 cells in a 2-mm cuvette and pulsed twice at 1.4 kV and 25 µF. Cells were seeded into 12-well plates or 25-cm^2^ flasks, and passaged at 3-to 4-day intervals posttransfection by trypsinization and reseeding with a 1∶3 to 1∶4 split into fresh culture vessels. When infectivity reached >90%, as was monitored by immunofluorescent staining with anti HCV core protein, cells were taken for cytotoxicity or substrate cleavage assays.

### Immunofluorescence Microscopy

1×10^5^ T-REx 293 cells inducibly expressing EGFP-scNS3 or EGFP-full NS3-4A were seeded on poly-L-lysine coated cover-slips in a 24 well-plate. After 12 hours, the cells were treated with 1µg/ml of tetracycline for 24 hours (or remained untreated), washed with PBS, fixed with 4% paraformaldehyde in PBS at room temperature for 20 minutes, permeabilized with Triton X-100 (0.1% in PBS) for 5 minutes, and blocked with 90% fetal calf serum/10% PBS at room temperature for 25 minutes. Slides were incubated with 1∶200 diluted rabbit-polyclonal anti-calnexin antibody (Sigma, USA) as primary antibody for 1 hour, and followed by 1∶500 diluted Cy3-conjugated anti-rabbit IgG (Jackson ImmunoResearch Laboratories, USA) secondary antibody and Hoechst 33258 (5µg/ml) (Sigma, USA) for 1 hour at room temperature. Slides were washed with PBS, mounted in ImmuGlo Mounting Medium (IMMCO Diagnostics, USA), and examined with a Zeiss LSM 510 META laser scanning confocal microscope.

For infectivity assays, Huh7.5 cells infected with HCV HJ3-5 chimeric virus were seeded into 8-well chamber slides (Nalge Nunc, USA). After 24 hours, cells were fixed and permeabilized as described above and stained with 1∶300 diluted mouse monoclonal antibody C7-50 (Affinity BioReagents, USA) specific for the HCV core protein followed by staining with 1∶100 diluted Cy2-conjugated goat anti-mouse IgG (Jackson ImmunoResearch Laboratories, USA). Slides were mounted and examined using a fluorescence microscope.

### *In-vitro* cleavage of DTA and RTA based toxins

600ng of DTA based toxins or 3000ng of RTA based toxins were incubated with or without 500ng or 1000ng, respectively, of recombinant MBP-scNS3 fusion [32] in a reaction buffer (50mM Tris-HCl (pH 7.5), 150mM NaCl, 0.05% tween 20, 20% glycerol and 1.7mM of DTT) in a total volume of 60µl for 1 hour at 37°C. NS3 mediated cleavage was verified by western blotting of 50ng (DTA based toxins) or 250ng (RTA based toxins) toxin samples using rabbit polyclonal anti-PE antibody as described above.

### ADP-ribosylation assay

The ADP-ribosylation activity of DTA based toxins was determined by measuring transfer of ADP-ribose from [^14^C]NAD to EF-2 essentially as described at [76]. Shortly, 30ng of each toxin were diluted to 210µL in 50mM Tris-HCl (pH 8.0), 1 mM EDTA, and 0.1% BSA. Mixture was incubated with wheat germ extract in the presence of 2.4 µM [^14^C] NAD (6×10^5^ cpm) (Amersham Biosciences, UK) for 40 minutes at RT. Reactions were terminated by addition of TCA to the reaction mixture which resulted in total protein precipitation. Level of ADP-ribosylated EF2 was assessed by measuring the radioactivity of the precipitated protein by a scintillation counter.

### Ribosome depurination assay

The catalytic activity of ricin A based toxins was determined by a modification of the *in-vitro* assay described at [53], [54]. A serial dilutions of the cleavable or uncleavable RTA based toxins (treated or untreated with NS3) were incubated with 10µl of micrococcal nuclease-treated rabbit reticulocyte lysate (Promega, USA) for 30 minutes at 30°C, after which total RNA from each mixture was extracted with phenol and chloroform, precipitated in ethanol and suspended in 22µl of water. Half of the RNA (11µl) was then treated with 50µl of acidic aniline (1M aniline in 2.8 M acetic acid) for 10 minutes at 40°C and the other half remained untreated. Next, RNA was recovered by precipitation with ammonium acetate and ethanol, and analyzed by 3% TBE agarose gel electrophoresis.

### Cell-viability assay

The Cell-killing activities of recombinant toxins were measured by a MTT assay. For cytotoxicity assay on T-REx 293 cells inducibly expressing NS3 protease: 4×10^4^ or 2×10^4^ cells were seeded per well in 96-well plates. After 9 or 24 hours, respectively, cells were treated with indicated concentration of tetracycline or left untreated. 2 or 8 hours later, respectively, cells were incubated with serial dilutions of the toxins (presence of tetracycline was kept in the growth media of induced cells). After 72 hours, the media was replaced by fresh media (100 µl per well) containing 1 mg/ml MTT (Thiazolyl Blue Tetrazoliam Bromide (Sigma, USA) dissolved in PBS) reagent and the cells were incubated for another 30 minutes. MTT-formazan crystals were dissolved by the addition of extraction solution (20% SDS, 50% DMF, pH 4.7) (100 µl per well) and incubation for 16 hours at 37°C. Absorbance at 570 nm was recorded on an automated microtiter plate reader. The results were expressed as percentage of living cells relatively to the untreated controls. The IC_50_ value is the concentration of the toxin which inhibited cell growth by 50%.

For cytotoxicity assay on HCV infected or uninfected HUH7.5 cells or NS3 expressing Huh7.5 cells: 1×10^4^ Huh7.5 cells uninfected or infected with HJ3-5 chimeric virus or stably expressing NS3 protease were seeded per well in 96-well plates. After 24 hours, cells were incubated with serial dilutions of the toxins. 96 hours later, the media was replaced by fresh media (100 µl per well) containing 1 mg/ml MTT and the cells were incubated for another 60 minutes. Further steps were identical to theses described above.

## Supporting Information

Table S1 Oligonucleotide primers used in the study.
(DOC)

Text S1 Protein sequences of NS3 cleavable and uncleavable zymoxins used in the study.
(DOC)
