# Supplementary material for: Engineered Toxins “Zymoxins” Are Activated by the HCV NS3 Protease by Removal of an Inhibitory Protein Domain
Source: PLoS One. 2011 Jan 14;6(1):e15916. doi: 10.1371/journal.pone.0015916 (PMC3021518; doi:10.1371/journal.pone.0015916)
Supplement: Table S1 — Oligonucleotide primers used in the study. (DOC) [file pone.0015916.s001.doc]

| **Primer name**  **Table S1.** Oligonucleotide primers used in the study. | **Sequence (5’ – 3’)** | **Orientation** | **Restriction sites underlined** | **Construct name** |
| --- | --- | --- | --- | --- |
| **1-cytons3** | TCGAGCTCAAGCTTCGCCACCATGGCGCCTATCGGCTCAGTAG | forward | *Hind*III | EGFP-scNS3 |
| **2- cytons3** | TGGATCCCGGGCCCTCTAGACTCGAGCGGCCGCCACTG | reverse | *Apa*I | EGFP-scNS3 |
| **3-DTAclv** | TTCCCCTCTAGAAATAATTTTGTTTAACTTTAAGAAGGAGATATACCATGAAAAAGACAGCTATCGCGATTG | forward | *Xba*I | PE-DTA-cleavage site-defensin |
| **4- DTAclv** | TCGAGCAGCACACGACATCCTCCGCTAGCTCCTGCGCGGCCGCTCCTTGCCCGGGCTGGCTGGCGTAGTC | reverse |  | PE-DTA-cleavage site-defensin |
| **5- DTAclv** | CGTCCTTGTGATGGTGATGGTGATGCTGCAGGCCAATTGCGTAGGACATCGAGCAGCACACGACATCCTC | reverse |  | PE-DTA-cleavage site-defensin |
| **6- DTAclv** | ACGGAGCTCGAATTCTTACAGCTCGTCCTTGTGATGGTGATGG | reverse | *Eco*RI | PE-DTA-cleavage site-defensin |
| **7-DTAclv** | CTCAGCTTCCTTTCGGGCTTT | reverse |  | PE-DTA-cleavage site-defensin |
| **8-DTAclv** | GGCCGCCTGTGGGCATTCTGCTGTGGTTCTGGTTCCCATCACCATCACCATCACAAGGACGAG | forward |  | PE-DTA-cleavage site-defensin |
| **9-DTAclv** | TGCATCGCGGGTGAACGTCGTTACGGCACTTGTATTTATCAAGGCCGCCTGTGGGCATTCTG | forward |  | PE-DTA-cleavage site-defensin |
| **10-DTAclv** | CGGTAGCGGCGGCTCCGGTGCGTGCTACTGTCGTATTCCGGCTTGCATCGCGGGTGAACGTCG | forward |  | PE-DTA-cleavage site-defensin |
| **11-DTAclv** | ATTGGCCTGCAGGGCGGTGGCGGTTCTGGCGGCGGCTCCGGTAGCGGCGGCTCCGG | forward | *Pst*I | PE-DTA-cleavage site-defensin |
| **12-DTAclv** | AAATTTGACGTCGGCACCGGTGGCGCTGATGATGTTGTTGATTCTTC | forward | *Aat*II | PE-DTA-cleavage site-defensin |
| **13-DTAclv** | AAATTTCAATTGCGTAGGACATCGAGCAGCACACGACATCCTCTGCACAGGCTTGAGCCATATACTCATAC | reverse | *Mfe*I | PE-DTA-cleavage site-defensin |
| **14-DTAunc** | AAATTTGACGTCGGCACCGGTGGCGCTGATGATGTTGTTGATTCTTC | forward | *Aat*II | PE-DTA-mutated cleavage site- defensin |
| **15-DTAunc** | TTGCCGCGGACATCGAGCGGCACACGACATCCTCTGCAC | reverse |  | PE-DTA-mutated cleavage site- defensin |
| **16-DTAunc** | ACCGCCCTGCAGGCCAATTGCCGCGGACATCGAGCG | reverse | *Pst*I | PE-DTA-mutated cleavage site- defensin |
| **17-DTAnoclv** | AAATTTGACGTCGGCACCGGTGGCGCTGATGATGTTGTTGATTCTTC | forward | *Aat*II | PE-DTA-no cleavage site- defensin |
| **18-DTAnoclv** | ACCGCCCTGCAGGCCAATTGCTGCACAGGCTTGAGCCATATAC | reverse | *Pst*I | PE-DTA-no cleavage site- defensin |
| **19-DTA2aclv** | CTCAGCTTCCTTTCGGGCTTT | reverse |  | PE-DTA-full 2a JFH1 cleavage site-defensin |
| **20-DTA2aclv** | GACCGTATGTTGCTCTATGAGCTACTCCTGGACTGGTGCGCTGGCAATTGGCCTGCAGGGCGGTG | forward |  | PE-DTA-full 2a JFH1 cleavage site-defensin |
| **21-DTA2aclv** | TATATGGCTCAGGCCTGTGCATCTGAAGAAGATGACACGACCGTATGTTGCTCTATGAGC | forward | *StuI* | PE-DTA-full 2a JFH1 cleavage site-defensin |
| **22-RTAclv** | AAATTTCCGCGGCATATTCCCCAAACAATACCC | forward | *Sac*II | PE-RTA-cleavage site-stalk peptide |
| **23-RTAclv** | AAATTTCAATTGCAAACTGTGACGATGGTGGAGG | reverse | *Mfe*I | PE-RTA-cleavage site-stalk peptide |
| **24-RTAclv** | AAATTTCCGCGGCATATTCCCCAAACAATACCC | forward | *Sac*II | PE-RTA-cleavage site-stalk peptide |
| **25-RTAclv** | CGTACGACATGCTACAACACACCACATCTTCGGATCCTGACGATGGTGGAGGTGCGC | reverse |  | PE-RTA-cleavage site-stalk peptide |
| **26-RTAclv** | CATGTCATCATCGGATTCTTCGGATTCCTCTGAGCCGCCACCGCCGTACGACATGCTACAACACA | reverse |  | PE-RTA-cleavage site-stalk peptide |
| **27-RTAclv** | GTCGGATTCCTCGGACTCCTCACCACCGGTGTCGAACAGACCAAAACCCATGTCATCATCGGATTCTTC | reverse |  | PE-RTA-cleavage site-stalk peptide |
| **28-RTAclv** | GTGATGCTGCAGAGAACCGGTGTCAAACAGGCCGAAGCCCATATCGTCGTCGGATTCCTCGGACTCCTC | reverse | *Pst*I | PE-RTA-cleavage site-stalk peptide |
| **29-RTAunc** | TCGTCAGGATCCGAGGATGTCGTGTGCCGCTCGATGTCCGCGGGCGGTGGCGGCTCAGAGGAATC | forward | *BamHI* | PE-RTA- mutated cleavage site-stalk peptide |
| **30-RTAunc** | ACGGAGCTCGAATTCTTACAGCTCGTCCTTGTGATGGTGATGG | reverse | *EcoRI* | PE-RTA- mutated cleavage site-stalk peptide |
| **31-RTA2aclv** | CTCAGCTTCCTTTCGGGCTTT | reverse |  | PE-RTA-full 2a JFH1 cleavage site- stalk peptide |
| **32-RTA2aclv** | TATGTTGCTCTATGAGCTACTCCTGGACTGGTGCGCTGGGCGGTGGCGGCTCAGAGG | forward |  | PE-RTA-full 2a JFH1 cleavage site- stalk peptide |
| **33-RTA2aclv** | CCATCGTCAGGATCCGAAGAAGATGACACGACCGTATGTTGCTCTATGAGCTACTCC | forward | *BamH*I | PE-RTA-full 2a JFH1 cleavage site- stalk peptide |
| **34-subful1b** | TAGAAGGCACAGTCGAGG | reverse |  | MBP-EGFP-full 1b NS5AB-CBD |
| **35-subful1b** | AAGACGTAGTATGTTGCTCTATGAGTTACACTTGGACCGGGACACCGGTATCAGGCAATTTGAAG | forward |  | MBP-EGFP-full 1b NS5AB-CBD |
| **36-subful1b** | CTACAAGGACGCTAGCGGTGCTGACACAGAAGACGTAGTATGTTGCTCTATG | forward | *Nhe*I | MBP-EGFP-full 1b NS5AB-CBD |
| **37-subful2a** | TAGAAGGCACAGTCGAGG | reverse |  | MBP-EGFP- full 2a JFH1 NS5AB-CBD |
| **38-subful2a** | TGACACGACCGTATGTTGCTCTATGAGCTACTCCTGGACTGGTGCGCTGACACCGGTATCAGGCAATTTG | forward |  | MBP-EGFP- full 2a JFH1 NS5AB-CBD |
| **39-subful2a** | ACAAGGACGCTAGCTCTGAAGAAGATGACACGACCGTATGTTGCTC | forward | *Nhe*I | MBP-EGFP- full 2a JFH1 NS5AB-CBD |
