# Supplementary material for: Engineered Toxins “Zymoxins” Are Activated by the HCV NS3 Protease by Removal of an Inhibitory Protein Domain
Source: PLoS One. 2011 Jan 14;6(1):e15916. doi: 10.1371/journal.pone.0015916 (PMC3021518; doi:10.1371/journal.pone.0015916)
Supplement: Text S1 — Protein sequences of NS3 cleavable and uncleavable zymoxins used in the study. (DOC) [file pone.0015916.s002.doc]

**Text S1**. Protein sequences of NS3 cleavable and uncleavable zymoxins used in the study and their color-highlighted key features.

PE-DTA-cleavage site-defensin:

MKKTAIAIAVALAGFATVAQAANLAEEAFDLWNECAKACVLDLKDGVRSSRMSVDPAIADTNGQGVLHYSMVLEGGNDALKLAIDNALSITSDGLTIRLEGGVEPNKPVRYSYTRQARGSWSLNWLVPIGHEKPSNIKVFIHELNAGNQLSHMSPIYTIEMGDELLAKLARDATFFVRAHESNEMQPTLAISHAGVSVVMAQTQPRREKRWSEWASGKVLCLLDPLDGVYNYLAQQRCNLDDTWEGKIYRVLAGNPAKHDLDIKPTVISHRLHFPEGGSLAALTAHQACHLPLETFTRHRQPRGWEQLEQCGYPVQRLVALYLAARLSWNQVDQVIRNALASPGSGGDLGEAIREQPEQARLALTLAAAESERFVRQGTGNDEAGAANADVVSLTCPVAAGECAGPADSGDALLERNYPTGAEFLGDGGDVGTGGADDVVDSSKSFVMENFSSYHGTKPGYVDSIQKGIQKPKSGTQGNYDDDWKGFYSTDNKYDAAGYSVDNENPLSGKAGGVVKVTYPGLTKVLALKVDNAETIKKELGLSLTEPLMEQVGTEEFIKRFGDGASRVVLSLPFAEGSSSVEYINNWEQAKALSVELEINFETRGKRGQDAMYEYMAQACAEDVVCCSMSYAIGLQGGGGSGGGSGSGGSGACYCRIPACIAGERRYGTCIYQGRLWAFCCGSGSHHHHHHKDEL

MKKTAIAIAVALAGFATVAQAANL : bacterial secretion signal

EDVVCCSMSY : P6-P4’ NS3 cleavable NS5A/B junction sequence derived from HCV 1b/1a genotype

PE-DTA-full 2a JFH1 cleavage site-defensin:

MKKTAIAIAVALAGFATVAQAANLAEEAFDLWNECAKACVLDLKDGVRSSRMSVDPAIADTNGQGVLHYSMVLEGGNDALKLAIDNALSITSDGLTIRLEGGVEPNKPVRYSYTRQARGSWSLNWLVPIGHEKPSNIKVFIHELNAGNQLSHMSPIYTIEMGDELLAKLARDATFFVRAHESNEMQPTLAISHAGVSVVMAQTQPRREKRWSEWASGKVLCLLDPLDGVYNYLAQQRCNLDDTWEGKIYRVLAGNPAKHDLDIKPTVISHRLHFPEGGSLAALTAHQACHLPLETFTRHRQPRGWEQLEQCGYPVQRLVALYLAARLSWNQVDQVIRNALASPGSGGDLGEAIREQPEQARLALTLAAAESERFVRQGTGNDEAGAANADVVSLTCPVAAGECAGPADSGDALLERNYPTGAEFLGDGGDVGTGGADDVVDSSKSFVMENFSSYHGTKPGYVDSIQKGIQKPKSGTQGNYDDDWKGFYSTDNKYDAAGYSVDNENPLSGKAGGVVKVTYPGLTKVLALKVDNAETIKKELGLSLTEPLMEQVGTEEFIKRFGDGASRVVLSLPFAEGSSSVEYINNWEQAKALSVELEINFETRGKRGQDAMYEYMAQACASEEDDTTVCCSMSYSWTGALAIGLQGGGGSGGGSGSGGSGACYCRIPACIAGERRYGTCIYQGRLWAFCCGSGSHHHHHHKDEL

MKKTAIAIAVALAGFATVAQAANL : bacterial secretion signal

SEEDDTTVCCSMSYSWTGAL : P10-P10’ NS3 cleavable NS5A/B junction sequence derived from HCV 2a genotype (strain JFH1)

PE-DTA-mutated cleavage site- defensin:

MKKTAIAIAVALAGFATVAQAANLAEEAFDLWNECAKACVLDLKDGVRSSRMSVDPAIADTNGQGVLHYSMVLEGGNDALKLAIDNALSITSDGLTIRLEGGVEPNKPVRYSYTRQARGSWSLNWLVPIGHEKPSNIKVFIHELNAGNQLSHMSPIYTIEMGDELLAKLARDATFFVRAHESNEMQPTLAISHAGVSVVMAQTQPRREKRWSEWASGKVLCLLDPLDGVYNYLAQQRCNLDDTWEGKIYRVLAGNPAKHDLDIKPTVISHRLHFPEGGSLAALTAHQACHLPLETFTRHRQPRGWEQLEQCGYPVQRLVALYLAARLSWNQVDQVIRNALASPGSGGDLGEAIREQPEQARLALTLAAAESERFVRQGTGNDEAGAANADVVSLTCPVAAGECAGPADSGDALLERNYPTGAEFLGDGGDVGTGGADDVVDSSKSFVMENFSSYHGTKPGYVDSIQKGIQKPKSGTQGNYDDDWKGFYSTDNKYDAAGYSVDNENPLSGKAGGVVKVTYPGLTKVLALKVDNAETIKKELGLSLTEPLMEQVGTEEFIKRFGDGASRVVLSLPFAEGSSSVEYINNWEQAKALSVELEINFETRGKRGQDAMYEYMAQACAEDVVCRSMSAAIGLQGGGGSGGGSGSGGSGACYCRIPACIAGERRYGTCIYQGRLWAFCCGSGSHHHHHHKDEL

MKKTAIAIAVALAGFATVAQAANL : bacterial secretion signal

EDVVCRSMSA : mutated uncleavable NS5A/B junction sequence derived from HCV 1b/1a genotype

PE-RTA-cleavage site-stalk peptide:

MKKTAIAIAVALAGFATVAQAANLAEEAFDLWNECAKACVLDLKDGVRSSRMSVDPAIADTNGQGVLHYSMVLEGGNDALKLAIDNALSITSDGLTIRLEGGVEPNKPVRYSYTRQARGSWSLNWLVPIGHEKPSNIKVFIHELNAGNQLSHMSPIYTIEMGDELLAKLARDATFFVRAHESNEMQPTLAISHAGVSVVMAQTQPRREKRWSEWASGKVLCLLDPLDGVYNYLAQQRCNLDDTWEGKIYRVLAGNPAKHDLDIKPTVISHRLHFPEGGSLAALTAHQACHLPLETFTRHRQPRGWEQLEQCGYPVQRLVALYLAARLSWNQVDQVIRNALASPGSGGDLGEAIREQPEQARLALTLAAAESERFVRQGTGNDEAGAANADVVSLTCPVAAGECAGPADSGDALLERNYPTGAEFLGDGGDVSFSTRGIFPKQYPIINFTTAGATVQSYTNFIRAVRGRLTTGADVRHEIPVLPNRVGLPINQRFILVELSNHAELSVTLALDVTNAYVVGYRAGNSAYFFHPDNQEDAEAITHLFTDVQNRYTFAFGGNYDRLEQLAGNLRENIELGNGPLEEAISALYYYSTGGTQLPTLARSFIICIQMISEAARFQYIEGEMRTRIRYNRRSAPDPSVITLENSWGRLSTAIQESNQGAFASPIQLQRRNGSKFSVYDVSILIPIIALMVYRCAPPPSSGSEDVVCCSMSYGGGGSEESEESDDDMGFGLFDTGGEESEESDDDMGFGLFDTGSLQHHHHHHKDEL

MKKTAIAIAVALAGFATVAQAANL : bacterial secretion signal

EDVVCCSMSY : P6-P4’ NS3 cleavable NS5A/B junction sequence derived from HCV 1b/1a genotype

PE-RTA-full 2a JFH1 cleavage site- stalk peptide:

MKKTAIAIAVALAGFATVAQAANLAEEAFDLWNECAKACVLDLKDGVRSSRMSVDPAIADTNGQGVLHYSMVLEGGNDALKLAIDNALSITSDGLTIRLEGGVEPNKPVRYSYTRQARGSWSLNWLVPIGHEKPSNIKVFIHELNAGNQLSHMSPIYTIEMGDELLAKLARDATFFVRAHESNEMQPTLAISHAGVSVVMAQTQPRREKRWSEWASGKVLCLLDPLDGVYNYLAQQRCNLDDTWEGKIYRVLAGNPAKHDLDIKPTVISHRLHFPEGGSLAALTAHQACHLPLETFTRHRQPRGWEQLEQCGYPVQRLVALYLAARLSWNQVDQVIRNALASPGSGGDLGEAIREQPEQARLALTLAAAESERFVRQGTGNDEAGAANADVVSLTCPVAAGECAGPADSGDALLERNYPTGAEFLGDGGDVSFSTRGIFPKQYPIINFTTAGATVQSYTNFIRAVRGRLTTGADVRHEIPVLPNRVGLPINQRFILVELSNHAELSVTLALDVTNAYVVGYRAGNSAYFFHPDNQEDAEAITHLFTDVQNRYTFAFGGNYDRLEQLAGNLRENIELGNGPLEEAISALYYYSTGGTQLPTLARSFIICIQMISEAARFQYIEGEMRTRIRYNRRSAPDPSVITLENSWGRLSTAIQESNQGAFASPIQLQRRNGSKFSVYDVSILIPIIALMVYRCAPPPSSGSEEDDTTVCCSMSYSWTGALGGGGSEESEESDDDMGFGLFDTGGEESEESDDDMGFGLFDTGSLQHHHHHHKDEL

MKKTAIAIAVALAGFATVAQAANL : bacterial secretion signal

SEEDDTTVCCSMSYSWTGAL : P10-P10’ NS3 cleavable NS5A/B junction sequence derived from HCV 2a genotype (strain JFH1)

PE-RTA- mutated cleavage site-stalk peptide:

MKKTAIAIAVALAGFATVAQAANLAEEAFDLWNECAKACVLDLKDGVRSSRMSVDPAIADTNGQGVLHYSMVLEGGNDALKLAIDNALSITSDGLTIRLEGGVEPNKPVRYSYTRQARGSWSLNWLVPIGHEKPSNIKVFIHELNAGNQLSHMSPIYTIEMGDELLAKLARDATFFVRAHESNEMQPTLAISHAGVSVVMAQTQPRREKRWSEWASGKVLCLLDPLDGVYNYLAQQRCNLDDTWEGKIYRVLAGNPAKHDLDIKPTVISHRLHFPEGGSLAALTAHQACHLPLETFTRHRQPRGWEQLEQCGYPVQRLVALYLAARLSWNQVDQVIRNALASPGSGGDLGEAIREQPEQARLALTLAAAESERFVRQGTGNDEAGAANADVVSLTCPVAAGECAGPADSGDALLERNYPTGAEFLGDGGDVSFSTRGIFPKQYPIINFTTAGATVQSYTNFIRAVRGRLTTGADVRHEIPVLPNRVGLPINQRFILVELSNHAELSVTLALDVTNAYVVGYRAGNSAYFFHPDNQEDAEAITHLFTDVQNRYTFAFGGNYDRLEQLAGNLRENIELGNGPLEEAISALYYYSTGGTQLPTLARSFIICIQMISEAARFQYIEGEMRTRIRYNRRSAPDPSVITLENSWGRLSTAIQESNQGAFASPIQLQRRNGSKFSVYDVSILIPIIALMVYRCAPPPSSGSEDVVCRSMSAGGGGSEESEESDDDMGFGLFDTGGEESEESDDDMGFGLFDTGSLQHHHHHHKDEL

MKKTAIAIAVALAGFATVAQAANL : bacterial secretion signal

EDVVCRSMSA : mutated uncleavable NS5A/B junction sequence derived from HCV 1b/1a genotype
